# Supplementary material for: Incidence and etiology of clinically-attended, antibiotic-treated diarrhea among children under five years of age in low- and middle-income countries: Evidence from the Global Enteric Multicenter Study
Source: PLoS Negl Trop Dis. 2020 Aug 10;14(8):e0008520. doi: 10.1371/journal.pntd.0008520 (PMC7444547; doi:10.1371/journal.pntd.0008520)
Supplement: S1 Text — The supporting information document includes further elaborations of methods (sections 1–5), 11 supplemental tables, and one supplemental figure, and citations to references in the supplemental text, as listed in the Table of Contents (page 1). (PDF) [file pntd.0008520.s001.pdf]

Supporting information

Incidence and etiology of clinically-attended, antibiotic-treated diarrhea among children under five years of age in low- and middle-income countries: evidence from the Global Enteric Multicenter Study

Joseph A. Lewnard, PhD, Elizabeth T. Rogawski McQuade, PhD, James A. Platts-Mills, MD, Karen L. Kotloff, MD, Ramanan Laxminarayan, PhD

Contents

1 Supplemental methods

Sec. 1: Descriptive analyses
Sec. 2: Attributable fraction estimation for clinically-attended, antibiotic-treated MSD and LSD.
Sec. 3: Incidence estimation for clinically-attended, antibiotic-treated MSD and LSD.
Sec. 4: Incidence and attributable fraction estimation for all clinically-attended, antibiotic-treated diarrhea.
Sec. 5: Pathogen associations with antibiotic treatment and other clinical factors

2 Supplemental tables

S1 Table: Quantitative molecular diagnostic data availability among clinically-attended, antibiotic-treated cases and controls in the primary study
S2 Table: Antibiotic treatment among diarrhea cases with and without other diagnoses
S3 Table: Incidence of clinically-attended diarrhea cases and clinically-attended, antibiotic-treated cases, by age and site
S4 Table: Multipliers for etiologic proportion estimates based on conventional diagnostic data
S5 Table: Adjusted attributable fractions of clinically-attended, antibiotic-treated moderate-to-severe diarrhea associated with individual pathogens, by age stratum and site, estimated from quantitative molecular diagnostic data
S6 Table: Adjusted attributable fractions of clinically-attended, antibiotic-treated less-severe diarrhea associated with individual pathogens, by age stratum and site, based on multipliers pooled across sites.
S7 Table: Adjusted attributable fractions of clinically-attended, antibiotic-treated less-severe diarrhea associated with individual pathogens, by age stratum and site, estimated from conventional diagnostic data
S8 Table: Adjusted attributable fractions of clinically-attended, antibiotic-treated moderate-to-severe diarrhea associated with individual pathogens, by age stratum and site, estimated from conventional diagnostic data
S9 Table: Adjusted attributable fractions of all clinically-attended, antibiotic-treated diarrhea associated with individual pathogens, by age stratum and site, estimated from conventional diagnostic data for both moderate-to-severe diarrhea and less-severe diarrhea
S10 Table: Comparison of adjusted attributable fractions estimates for individual pathogens in clinically-attended, antibiotic-treated moderate-to-severe diarrhea with and without verified antibiotic administration
S11 Table: Data sources

3 Supplemental figures

S1 Figure: Multipliers for etiologic fractions based on site-specific estimates

4 Supplemental references

S1 DESCRIPTIVE ANALYSES

Our analysis included children with MSD in the primary and follow-on studies, and children with LSD in the follow-on study only, as well as their matched controls. We computed the weighted proportion of cases within each site and age stratum receiving antibiotics, defining weights as the inverse of the sampling fraction for cases (i.e., the number of eligible cases divided by the number of enrolled cases) over 16-week periods for each site, consistent with primary analyses, so that each 16-week period would receive equal weight.[1,2] We stratified estimates of the proportion of MSD cases and LSD cases receiving antibiotics; for the presentation of data in **Table 1** and **Table S2**, we further stratified proportions of dysenteric and non-dysenteric MSD cases receiving antibiotics.

We used the same approach to estimate the proportion of children with clinically-attended, antibiotic-treated MSD and LSD receiving each of the following antibiotics or antibiotic classes (**Figure 1**): trimethoprim/sulfamethoxazole, quinolones, penicillins, gentamicin, chloramphenicol, azithromycin, other macrolides, and other antibiotics. Because each child could receive multiple antibiotic treatments, estimates do not necessarily sum to 100%. We computed 95% confidence intervals around proportions via bootstrap resampling.

S2 ATTRIBUTABLE FRACTION ESTIMATION FOR CLINICALLY-ATTENDED, ANTIBIOTIC-TREATED MSD AND LSD

S2.1 General overview. We estimated adjusted attributable fractions associated with each pathogen for each site and age stratum using the general frameworks applied in previous analyses of the GEMS datasets.[1–4] Because use of conventional diagnostic assays has been found to under-estimate the burden of certain enteric pathogens,[4,5] we sought to correct for bias associated with the use of conventional diagnostic data in estimating adjusted attributable fractions of clinically-attended, antibiotic-treated LSD associated with each pathogen. Both conventional microbiological data and quantitative molecular diagnostic data (qPCR cycle quantification [Cq] number) were available for MSD cases and their matched controls. We used the ratio of adjusted attributable fraction estimates based on qPCR and conventional datasets for clinically-attended, antibiotic-treated MSD to re-scale our estimates of this same parameter for clinically-attended, antibiotic-treated LSD, where only conventional diagnostic data were available. We used two distinct frameworks to estimate adjusted attributable fractions based on conventional and quantitative microbiological data, as detailed below (*S2.2-2.3*). We describe the estimation of scaling factors to relate estimates based on conventional diagnostic data and qPCR data in *S2.4*.

S2.2 Attributable fraction estimation from conventional diagnostic data. For analyses of conventional diagnostic data, we fitted conditional logistic regression models defining clinically-attended, antibiotic-treated MSD or LSD versus control status as the outcome, and detection of each pathogen as covariates; models further accounted for matching strata. We did stepwise backward elimination of covariates from models that initially included all two-way interactions among pathogens, and between pathogens and age strata;[3] we arrived at final model specifications by sequentially excluding covariates and interactions until all met a p<0.05 cutoff. We estimated the adjusted attributable fraction associated with each pathogen (or pathogen combination) ℓ by multiplying its weighted prevalence among cases by $1 - 1/OR_{\ell}$, as estimated using the conditional logistic regression models. Weights were defined as the inverse of the sampling fraction for cases, as described above (*section S1*). We generated median estimates and 95% confidence intervals across 1000 bootstrap iterations of this procedure for each setting and age group.

S2.3 Attributable fraction estimation from quantitative molecular diagnostic data. For analyses of quantitative molecular diagnostic data, we fitted conditional logistic regression models that defined clinically-attended, antibiotic-treated MSD versus control status as the outcome; models included linear and quadratic Cq values for each pathogen as covariates, interacted with age strata, and accounted for matching strata. We did not undertake additional variable selection; in contrast to previous analyses, we did not include random effects for site.[4] We estimated adjusted attributable fractions by summing pathogen attributions across each of j cases using the formula

$$AF_i = \frac{\sum_{i=1}^{j} \omega_i \left(1 - \frac{1}{OR_i}\right)}{\sum_{i=1}^{j} \omega_i},$$

where OR_i indicated the relative odds of case status given a pathogen's Cq value for the ith child, and ω_i indicated the weight of the ith child (defined as the inverse of the sampling fraction, as defined above in *section S1*). Again, we generated median estimates and 95% confidence intervals across 1000 bootstrap iterations of this procedure for each setting and age group.

S2.4 Combining data based on multiple diagnostic assays. Defining $AF_\ell^C(k|\text{MSD})$ and $AF_\ell^Q(k|\text{MSD})$ as our estimates for the fraction of clinically-attended, antibiotic-treated MSD attributable to pathogen ℓ in age group *k*, based on conventional and quantitative molecular diagnostic data, respectively, we estimated

$$w_\ell(k|\text{MSD}) = \frac{AF_\ell^Q(k|\text{MSD})}{AF_\ell^C(k|\text{MSD})}$$

as a multiplier indicating the ratio of adjusted attributable fraction estimates based on qPCR data and conventional diagnostic data. We used adjusted attributable fraction estimates aggregated across sites (excluding Kenya) to generate $w_\ell(k|\text{MSD})$ estimates (**Table S4**). Further stratification by site led to unstable estimates, in particular where analyses were underpowered to supply statistically-significant odds ratio estimates for individual pathogens within each site and age group (**Figure S1**).

We defined $AF_\ell^{\hat{Q}}(k|\text{LSD})$ as the estimate of the fraction of clinically-attended, antibiotic-treated LSD attributable to pathogen ℓ in age group *k* that we would expect to obtain if quantitative molecular diagnostic data were available, given our estimates from conventional diagnostic data, $AF_\ell^C(k|\text{LSD})$. We estimated this quantity as

$$AF_\ell^{\hat{Q}}(k|\text{LSD}) = w_\ell(k|\text{MSD}) \times AF_\ell^C(k|\text{LSD}),$$

assuming the ratio $w_\ell(k|\text{MSD})$ of estimates based on differing diagnostic assays in clinically-attended, antibiotic-treated MSD cases would provide the best approximation of the same ratio in clinically-attended, antibiotic-treated LSD cases. We present adjusted attributable fraction estimates for clinically-attended, antibiotic-treated MSD and LSD based on conventional diagnostic data in **Tables S7**-**S8**. We present pooled, adjusted attributable fraction estimates for all clinically-attended, antibiotic-treated diarrhea based on conventional diagnostic data in **Table S9**.

Though we interpret AF_ℓ^C, AF_ℓ^Q, and $AF_\ell^{\hat{Q}}$ as proportions, these estimators were not strictly bounded on [0,1] due to the continuous nature of the odds ratio estimate. We used the full distribution of our AF_ℓ^C and AF_ℓ^Q estimates (potentially below zero or greater than one) to define $w_\ell(k)$ multipliers. Because proportion estimates below zero or above one do not have a natural interpretation, we report values of AF_ℓ^C, AF_ℓ^Q, and $AF_\ell^{\hat{Q}}$ below zero or above one as zero or one, respectively. Thus, inclusion of 0% in the 95% confidence interval can be interpreted to indicate a pathogen is not significantly associated with clinically-attended, antibiotic-treated MSD or LSD at the two-sided $p<0.05$ threshold; inclusion of 100% in the confidence interval can be interpreted to indicate that the results do not exclude the possibility that all cases are attributable to a given pathogen, at the two-sided $p<0.05$ threshold.

S3 INCIDENCE ESTIMATION FOR CLINICALLY-ATTENDED, ANTIBIOTIC-TREATED MSD AND LSD

Data from serial healthcare attitudes and utilization surveys provide an estimate of $M_{\text{SHC}}^{\text{MSD}}$ and $M_{\text{SHC}}^{\text{LSD}}$, the number of acute MSD or LSD cases taken to sentinel hospital or health center within seven days of onset during the study period across *N* child-years at risk in the demographic surveillance site population of

each setting, as well as r^{MSD} and r^{LSD}, the proportion of all incident MSD or LSD cases taken to a sentinel hospital or health center within seven days of disease onset.[3] Whereas primary analyses of the GEMS datasets used the formulations $M_{\mathrm{SHC}}^{\mathrm{MSD}}/(r^{\mathrm{MSD}} \times N)$ and $M_{\mathrm{SHC}}^{\mathrm{LSD}}/(r^{\mathrm{LSD}} \times N)$ to estimate total MSD and LSD incidence, respectively, we could not assume the likelihood of antibiotic treatment was equal among cases that did and did not seek care.

We therefore estimated the incidence rates of clinically-attended, antibiotic-treated MSD and LSD cases in each age group, *k* ($\Lambda^{\mathrm{MSD}}(k)$ and $\Lambda^{\mathrm{LSD}}(k)$, respectively), as

$$\Lambda^{\mathrm{MSD}}(k) = M_{\mathrm{SCH}}^{\mathrm{MSD}}(k) \times \Pr(\mathrm{Antibiotic}|\mathrm{MSD\ Case}, k)\ /N(k)$$

and

$$\Lambda^{\mathrm{LSD}}(k) = M_{\mathrm{SCH}}^{\mathrm{LSD}}(k) \times \Pr(\mathrm{Antibiotic}|\mathrm{LSD\ Case}, k)\ /N(k).$$

S4 INCIDENCE AND ADJUSTED ATTRIBUTABLE FRACTION ESTIMATION FOR ALL CLINICALLY-ATTENDED, ANTIBIOTIC-TREATED DIARRHEA

We aimed to estimate total incidence and pathogen-specific adjusted attributable fractions accounting for antibiotic use associated with both MSD and LSD cases. We defined the incidence of all clinically-attended, antibiotic-treated diarrhea as $\Lambda^{\mathrm{All}}(k) = \Lambda^{\mathrm{MSD}}(k) + \Lambda^{\mathrm{LSD}}(k)$. We estimated the incidence all clinically-attended, antibiotic-treated attributable to each pathogen *i*, within each age group *k*, as

$$\lambda_i^{\mathrm{All}}(k) = AF_i^{Q}(k|\mathrm{MSD})\Lambda^{\mathrm{MSD}} + AF_i^{\hat{Q}}(k|\mathrm{LSD})\Lambda^{\mathrm{LSD}}.$$

We estimated the fraction of all clinically-attended, antibiotic-treated diarrhea attributable to each pathogen *i*, within each age group *k*, as $\lambda_i^{\mathrm{All}}(k)/\Lambda^{\mathrm{All}}(k)$.

We repeated the analyses described in sections *S2-S4* for both dysenteric and non-dysenteric MSD endpoints in order to estimate the incidence of clinically-attended, antibiotic-treated associated with dysenteric and non-dysenteric *Shigella*-attributable MSD. We summed non-dysenteric *Shigella*-attributable clinically-attended, antibiotic-treated incidence as *Shigella*-attributable non-dysenteric clinically-attended, antibiotic-treated MSD plus *Shigella*-attributable clinically-attended, antibiotic-treated LSD.

S5 PATHOGEN ASSOCIATIONS WITH ANTIBIOTIC TREATMENT AND OTHER CLINICAL FACTORS

We also sought to determine the association of each pathogen with the likelihood of receiving of antibiotics (given MSD or LSD presentation), becoming hospitalized (for MSD cases), and experiencing dysentery (for MSD cases). Using conditional logistic regression models, we estimated adjusted ORs of detection of each pathogen among antibiotic-treated cases versus those not treated with antibiotics (for each of MSD and LSD cases), hospitalized MSD cases versus those not hospitalized, and dysentery cases versus non-dysenteric MSD cases. We stratified cases by site, fortnight, and age (by month of life). For analyses of MSD data, we defined pathogen detection as Cq<35 and absence as Cq≥35. We excluded children diagnosed with pneumonia/lower respiratory tract infection, meningitis or other invasive disease, or typhoid, and controlled for the occurrence of any other non-diarrhea diagnosis in regression models.

S1 Table: Quantitative molecular diagnostic data coverage among clinically-attended, antibiotic-treated cases and controls in the primary study.

Outcome	Measure	Setting						
		The Gambia	Mali	Mozambique	Kenya	India	Bangladesh	Pakistan
Ages 6 weeks to 11 months								
MSD with dysentery								
	TaqMan array performed, *N*	22	4	15	6	18	211	40
	Administered or prescribed antibiotics, *n*	21	4	13	6	18	211	33
MSD without dysentery								
	TaqMan array performed, *N*	281	298	251	301	269	88	266
	Administered or prescribed antibiotics, *n*	206	280	211	234	239	69	18
Controls								
	TaqMan array performed, *N*	298	296	261	297	286	298	295
Ages 12 to 23 months								
MSD with dysentery								
	TaqMan array performed, *N*	55	18	30	7	27	213	56
	Administered or prescribed antibiotics, *n*	53	18	28	7	25	212	49
MSD without dysentery								
	TaqMan array performed, *N*	253	284	127	295	273	87	242
	Administered or prescribed antibiotics, *n*	194	263	104	226	259	65	16
Controls								
	TaqMan array performed, *N*	299	299	157	294	294	295	299
Ages 24 to 59 months								
MSD with dysentery								
	TaqMan array performed, *N*	34	18	29	25	57	230	63
	Administered or prescribed antibiotics, *n*	32	17	28	23	55	229	58
MSD without dysentery								
	TaqMan array performed, *N*	128	264	60	269	239	67	162
	Administered or prescribed antibiotics, *n*	104	244	46	209	229	53	11
Controls								
	TaqMan array performed, *N*	161	281	93	280	291	298	225

Abbreviations: MSD: moderate-to-severe diarrhea

Numbers in the table indicate the total sample of participants experiencing dysenteric MSD and non-dysenteric MSD, and control children, whose samples were selected for processing via TaqMan array cards for enteropathogen quantification.

S2 Table: Antibiotic treatment among diarrhea cases with and without other diagnoses.

Setting and age group	Definitions	Total cases	Cases with other non-diarrhea diagnoses justifying antibiotic treatment				Cases with additional non-diarrhea diagnoses	
			Pneumonia/lower respiratory infection	Meningitis	Other invasive disease	Typhoid	Malaria	Other
The Gambia								
6 weeks to 11 months								
	All MSD with dysentery, *N*	52	0	0	5	0	13	2
	CAAT-MSD with dysentery, *n* (%)	49 (94.2)	0	0	5 (100)	0	11 (86.46)	2 (100)
	All MSD without dysentery, *N*	472	48	3	32	0	192	32
	CAAT-MSD without dysentery, *n* (%)	376 (79.7)	47 (97.9)	3 (100)	30 (93.8)	0	145 (75.5)	21 (65.6)
	All LSD, *N*	220	9	0	5	0	2	6
	CAAT-LSD, *n* (%)	181 (82.3)	8 (88.9)	0	5 (100)	0	1 (50.0)	6 (100)
12-23 months								
	All MSD with dysentery, *N*	137	8	0	2	0	22	1
	CAAT-MSD with dysentery, *n* (%)	122 (89.1)	7 (87.5)	0	2 (100)	0	21 (95.5)	1 (100)
	All MSD without dysentery, *N*	477	45	0	16	0	218	39
	CAAT-MSD without dysentery, *n* (%)	382 (80.1)	41 (91.1)	0	16 (100)	0	168 (77.1)	27 (69.2)
	All LSD, *N*	202	7	0	3	0	6	8
	CAAT-LSD, *n* (%)	162 (80.2)	7 (100)	0	2 (66.7)	0	4 (66.7)	4 (50.0)
24-59 months								
	All MSD with dysentery, *N*	70	2	1	0	0	20	2
	CAAT-MSD with dysentery, *n* (%)	64 (91.4)	2 (100)	1 (100)	0	0	18 (90.0)	2 (100)
	All MSD without dysentery, *N*	173	8	1	3	0	73	12
	CAAT-MSD without dysentery, *n* (%)	143 (82.7)	8 (100)	1 (100)	3 (100)	0	57 (78.1)	9 (75.0)
	All LSD, *N*	135	6	0	2	0	5	6
	CAAT-LSD, *n* (%)	113 (83.7)	6 (100)	0	2 (100)	0	3 (60.0)	6 (100)
Mali								
6 weeks to 11 months								
	All MSD with dysentery, *N*	23	0	0	0	0	4	0
	CAAT-MSD with dysentery, *n* (%)	22 (95.7)	0	0	0	0	4 (100)	0
	All MSD without dysentery, *N*	938	81	2	3	2	351	72
	CAAT-MSD without dysentery, *n* (%)	869 (92.6)	81 (100)	2 (100)	2 (66.7)	1 (50.0)	338 (96.3)	71 (98.6)
	All LSD, *N*	236	3	0	0	0	45	16
	CAAT-LSD, *n* (%)	197 (83.5)	3 (100)	0	0	0	44 (97.8)	16 (100)
12-23 months								
	All MSD with dysentery, *N*	50	2	0	0	0	19	0
	CAAT-MSD with dysentery, *n* (%)	49 (98.0)	2 (100)	0	0	0	19 (100)	0
	All MSD without dysentery, *N*	861	77	0	3	1	340	43
	CAAT-MSD without dysentery, *n* (%)	805 (93.5)	77 (100)	0	3 (100)	1 (100)	331 (97.4)	42 (97.7)
	All LSD, *N*	226	2	0	0	0	43	14
	CAAT-LSD, *n* (%)	196 (86.7)	2 (100)	0	0	0	40 (93.0)	14 (100)
24-59 months								
	All MSD with dysentery, *N*	61	2	0	0	0	18	2
	CAAT-MSD with dysentery, *n* (%)	57 (93.4)	2 (100)	0	0	0	18 (100)	2 (100)
	All MSD without dysentery, *N*	784	65	0	0	0	251	26
	CAAT-MSD without dysentery, *n* (%)	731 (93.2)	63 (96.9)	0	0	0	246 (98.0)	26 (100)
	All LSD, *N*	230	1	0	0	0	15	6
	CAAT-LSD, *n* (%)	175 (76.1)	1 (100)	0	0	0	15 (100)	6 (100)
Mozambique								
6 weeks to 11 months								
	All MSD with dysentery, *N*	28	2	0	0	0	2	5
	CAAT-MSD with dysentery, *n* (%)	23 (82.1)	2 (100)	0	0	0	2 (100)	5 (100)
	All MSD without dysentery, *N*	411	88	1	4	0	74	150
	CAAT-MSD without dysentery, *n* (%)	335 (81.5)	84 (95.5)	1 (100)	4 (100)	0	42 (56.8)	133 (88.7)
	All LSD, *N*	155	5	0	0	0	9	23
	CAAT-LSD, *n* (%)	83 (53.5)	5 (100)	0	0	0	0 (0.0)	16 (69.6)

12-23 months								
	All MSD with dysentery, *N*	45	3	0	0	0	2	2
	CAAT-MSD with dysentery, *n* (%)	41 (91.1)	3 (100)	0	0	0	2 (100)	2 (100)
	All MSD without dysentery, *N*	193	44	0	1	0	36	72
	CAAT-MSD without dysentery, *n* (%)	151 (78.2)	41 (93.2)	0	1 (100)	0	21 (58.3)	59 (81.9)
	All LSD, *N*	175	4	0	0	0	13	24
	CAAT-LSD, *n* (%)	95 (54.3)	4 (100)	0	0	0	0 (0)	17 (70.8)
24-59 months								
	All MSD with dysentery, *N*	53	1	0	0	0	6	4
	CAAT-MSD with dysentery, *n* (%)	47 (88.7)	1 (100)	0	0	0	4 (66.70	3 (75.0)
	All MSD without dysentery, *N*	84	9	0	1	0	22	42
	CAAT-MSD without dysentery, *n* (%)	61 (72.6)	9 (100)	0	1 (100)	0	10 (45.5)	37 (88.1)
	All LSD, *N*	101	3	0	0	0	12	17
	CAAT-LSD, *n* (%)	53 (52.5)	3 (100)	0	0	0	1 (8.3)	8 (47.1)
Kenya								
6 weeks to 11 months								
	All MSD with dysentery, *N*	23	2	0	2	0	6	20
	CAAT-MSD with dysentery, *n* (%)	23 (100)	2 (100)	0	2 (100)	0	6 (100)	20 (100)
	All MSD without dysentery, *N*	806	72	1	44	0	440	787
	CAAT-MSD without dysentery, *n* (%)	574 (71.2)	71 (98.6)	1 (100)	42 (95.5)	0	302 (68.6)	557 (70.8)
	All LSD, *N*	– –	– –	– –	– –	– –	– –	– –
	CAAT-LSD, *n* (%)	– –	– –	– –	– –	– –	– –	– –
12-23 months								
	All MSD with dysentery, *N*	21	0	0	0	0	14	17
	CAAT-MSD with dysentery, *n* (%)	20 (95.2)	0	0	0	0	14 (100)	16 (94.1)
	All MSD without dysentery, *N*	470	32	0	19	0	287	458
	CAAT-MSD without dysentery, *n* (%)	342 (72.8)	31 (96.9)	0	19 (100)	0	203 (70.7)	330 (72.1)
	All LSD, *N*	– –	– –	– –	– –	– –	– –	– –
	CAAT-LSD, *n* (%)	– –	– –	– –	– –	– –	– –	– –
24-59 months								
	All MSD with dysentery, *N*	42	1	0	4	0	27	37
	CAAT-MSD with dysentery, *n* (%)	39 (92.9)	1 (100)	0	4 (100)	0	25 (92.6)	34 (91.9)
	All MSD without dysentery, *N*	416	31	0	12	0	273	398
	CAAT-MSD without dysentery, *n* (%)	302 (72.6)	31 (100)	0	12 (100)	0	195 (71.4)	285 (71.6)
	All LSD, *N*	– –	– –	– –	– –	– –	– –	– –
	CAAT-LSD, *n* (%)	– –	– –	– –	– –	– –	– –	– –
India								
6 weeks to 11 months								
	All MSD with dysentery, *N*	63	0	0	0	0	0	9
	CAAT-MSD with dysentery, *n* (%)	59 (86.5)	0	0	0	0	0	9 (100)
	All MSD without dysentery, *N*	815	4	0	0	0	0	126
	CAAT-MSD without dysentery, *n* (%)	723 (88.7)	4 (100)	0	0	0	0	116 (92.1)
	All LSD, *N*	213	0	0	0	0	0	31
	CAAT-LSD, *n* (%)	79 (37.1)	0	0	0	0	0	26 (83.9)
12-23 months								
	All MSD with dysentery, *N*	80	0	0	0	0	0	9
	CAAT-MSD with dysentery, *n* (%)	77 (96.2)	0	0	0	0	0	9 (100)
	All MSD without dysentery, *N*	672	4	0	1	0	0	112
	CAAT-MSD without dysentery, *n* (%)	637 (94.8)	4 (100)	0	1 (100)	0	0	109 (97.3)
	All LSD, *N*	180	0	0	0	0	0	38
	CAAT-LSD, *n* (%)	92 (51.1)	0	0	0	0	0	34 (89.5)
24-59 months								
	All MSD with dysentery, *N*	98	1	0	0	0	0	13
	CAAT-MSD with dysentery, *n* (%)	96 (98.0)	1 (100)	0	0	0	0	13 (100)
	All MSD without dysentery, *N*	379	0	0	0	0	1	79
	CAAT-MSD without dysentery, *n* (%)	364 (96.0)	0	0	0	0	1 (100)	78 (98.7)
	All LSD, *N*	181	1	0	0	0	0	36
	CAAT-LSD, *n* (%)	108 (59.7)	0	0	0	0	0	30 (83.3)

Bangladesh								
6 weeks to 11 months								
	All MSD with dysentery, *N*	500	0	0	0	0	0	23
	CAAT-MSD with dysentery, *n* (%)	497 (99.4)	0	0	0	0	0	23 (100)
	All MSD without dysentery, *N*	172	0	0	0	0	0	31
	CAAT-MSD without dysentery, *n* (%)	137 (79.7)	0	0	0	0	0	25 (64.5)
	All LSD, *N*	183	0	0	0	0	0	0
	CAAT-LSD, *n* (%)	110 (60.1)	0	0	0	0	0	0
12-23 months								
	All MSD with dysentery, *N*	420	0	0	0	0	0	25
	CAAT-MSD with dysentery, *n* (%)	418 (99.5)	0	0	0	0	0	25 (100)
	All MSD without dysentery, *N*	159	0	0	0	0	0	33
	CAAT-MSD without dysentery, *n* (%)	122 (76.7)	0	0	0	0	0	25 (75.8)
	All LSD, *N*	148	0	0	0	0	0	0
	CAAT-LSD, *n* (%)	96 (64.9)	0	0	0	0	0	0
24-59 months								
	All MSD with dysentery, *N*	363	0	0	0	0	0	26
	CAAT-MSD with dysentery, *n* (%)	361 (99.4)	0	0	0	0	0	25 (96.2)
	All MSD without dysentery, *N*	100	0	0	0	1	0	28
	CAAT-MSD without dysentery, *n* (%)	76 (76.0)	0	0	0	1 (100)	0	20 (71.4)
	All LSD, *N*	83	0	0	0	0	0	0
	CAAT-LSD, *n* (%)	50 (60.2)	0	0	0	0	0	0
Pakistan								
6 weeks to 11 months								
	All MSD with dysentery, *N*	106	4	0	0	0	0	5
	CAAT-MSD with dysentery, *n* (%)	91 (85.8)	3 (75.0)	0	0	0	0	5 (100)
	All MSD without dysentery, *N*	682	64	0	2	0	0	40
	CAAT-MSD without dysentery, *n* (%)	65 (9.5)	18 (28.1)	0	1 (50.0)	0	0	8 (20.0)
	All LSD, *N*	227	11	0	1	0	0	27
	CAAT-LSD, *n* (%)	26 (11.5)	2 (18.2)	0	0 (0.0)	0	0	9 (33.3)
12-23 months								
	All MSD with dysentery, *N*	122	0	0	0	0	0	6
	CAAT-MSD with dysentery, *n* (%)	104 (85.2)	0	0	0	0	0	5 (83.3)
	All MSD without dysentery, *N*	390	21	1	2	0	0	21
	CAAT-MSD without dysentery, *n* (%)	46 (11.8)	5 (23.8)	1 (100)	0 (0.0)	0	0	6 (28.6)
	All LSD, *N*	171	4	0	1	0	0	19
	CAAT-LSD, *n* (%)	18 (10.5)	2 (50.0)	0	1 (100)	0	0	4 (21.1)
24-59 months								
	All MSD with dysentery, *N*	97	1	0	0	0	0	3
	CAAT-MSD with dysentery, *n* (%)	86 (88.7)	1 (100)	0	0	0	0	2 (66.7)
	All MSD without dysentery, *N*	201	11	0	2	0	0	8
	CAAT-MSD without dysentery, *n* (%)	20 (10.0)	4 (36.4)	0	0 (0.0)	0	0	0 (0.0)
	All LSD, *N*	108	1	0	0	0	0	12
	CAAT-LSD, *n* (%)	10 (9.3)	0	0	0	0	0	5 (41.7)

Abbreviations: MSD: moderate-to-severe diarrhea; LSD: less-severe diarrhea; CAAT: Clinically-attended, antibiotic-treated

Percentages indicate the proportion of cases administered antibiotics or receiving an antibiotic prescription (for which antibiotic administration could not be verified) for each of dysenteric MSD, non-dysenteric MSD, and LSD. We considered pneumonia/lower respiratory infection, meningitis or other invasive infection, and typhoid diagnoses to warrant antibiotic treatment irrespective of diarrhea symptoms; thus, cases receiving antibiotics but diagnosed with any of these conditions were excluded from the definition of clinically-attended, antibiotic-treated diarrhea.

S3 Table: Incidence of clinically-attended diarrhea cases and clinically-attended, antibiotic-treated cases, by age and site.

Outcome	Measure	Setting						
		The Gambia	Mali	Mozambique	India	Bangladesh	Pakistan	All (excl. Kenya)
Ages 6 weeks to 11 months								
Clinically-attended diarrhea	Incidence rate, per 100	22.4 (11.1, 42.6)	12.7 (6.2, 28.4)	21.5 (6.9, 72.8)	30.6 (19.8, 46.0)	10.7 (3.9, 44.4)	34.1 (20.4, 55.0)	23.4 (17.3, 35.3)
Clinically-attended, antibiotic-treated diarrhea	Incidence rate, per 100	16.6 (8.0, 31.8)	10.6 (5.5, 22.3)	11.2 (3.8, 40.3)	17.4 (11.3, 25.4)	8.2 (3.5, 27.7)	4.1 (2.4, 7.3)	12.2 (9.0, 17.8)
Ages 12 to 23 months								
Clinically-attended diarrhea	Incidence rate, per 100	18.1 (9.8, 32.0)	8.8 (3.5, 22.5)	19.5 (7.1, 54.1)	22.5 (13.0, 39.4)	8.3 (3.5, 19.8)	28.3 (13.8, 55.1)	18.7 (13.6, 26.1)
Clinically-attended, antibiotic-treated diarrhea	Incidence rate, per 100	13.8 (7.6, 24.9)	7.7 (2.9, 19.9)	10.7 (4.0, 27.2)	16.0 (9.6, 25.2)	6.3 (2.8, 14.3)	3.2 (1.6, 6.0)	10.2 (7.4, 13.9)
Ages 24 to 59 months								
Clinically-attended diarrhea	Incidence rate, per 100	2.3 (1.1, 4.5)	2.1 (0.5, 6.5)	3.3 (0.8, 11.7)	5.2 (2.5, 9.8)	1.2 (0.3, 5.4)	5.5 (2.4, 11.9)	3.6 (2.4, 5.6)
Clinically-attended, antibiotic-treated diarrhea	Incidence rate, per 100	1.8 (0.9, 3.5)	1.7 (0.4, 5.3)	1.8 (0.4, 6.0)	3.6 (1.7, 7.5)	1.0 (0.2, 3.5)	0.7 (0.3, 1.6)	1.9 (1.3, 3.0)
Ages 6 weeks to 59 months								
Clinically-attended diarrhea	Incidence rate, per 100	9.8 (6.6, 14.4)	5.9 (3.7, 10.7)	11.4 (5.5, 24.0)	14.1 (10.5, 18.9)	5.0 (2.6, 12.4)	16.2 (11.2, 23.5)	10.7 (8.7, 13.4)
Clinically-attended, antibiotic-treated diarrhea	Incidence rate, per 100	7.4 (4.8, 10.8)	5.0 (3.0, 8.8)	6.1 (3.1, 12.6)	9.1 (6.6, 12.3)	3.7 (2.1, 7.8)	2.0 (1.4, 2.9)	5.7 (4.6, 7.2)

Incidence estimates (per 100 child-years at risk) listed in this table are plotted in **Figure 3** for each setting. We present overall pathogen-specific incidence rates for clinically-attended, antibiotic-treated diarrhea, across all settings, in **Figure 4**. Estimated incidence rates exclude diarrhea cases receiving antibiotics who were also diagnosed with pneumonia/lower respiratory tract infection, meningitis or other invasive disease, or typhoid, as these conditions warrant antibiotic treatment irrespective of diarrhea symptoms.

S4 Table: Multipliers for etiologic proportion estimates based on conventional diagnostic data.

Pathogen	Age group		
	6 weeks to 11m	12-23m	24-59m
Shigella	2.24 (1.73, 2.88)	2.61 (2.35, 2.91)	2.39 (2.14, 2.69)
Rotavirus	1.69 (1.48, 1.89)	1.86 (1.59, 2.15)	2.17 (1.63, 2.82)
Adenovirus 40/41	9.23 (6.35, 15.85)	8.38 (5.16, 18.73)	6.53 (0.00, 22.22)
C. jejuni	3.00 (1.61, 5.30)	2.05 (0.10, 4.84)	3.84 (0.88, 7.88)
Cryptosporidium	0.96 (0.73, 1.29)	0.92 (0.56, 1.57)	– –
ST-ETEC (ST only or LT/ST)	2.64 (1.59, 4.06)	1.86 (1.34, 2.69)	1.13 (0.62, 1.90)
Norovirus GII	2.74 (1.43, 7.51)	1.42 (0.47, 7.33)	1.63 (0.76, 3.17)
Sapovirus	8.44 (0.00, 81.90)	6.67 (0.00, 65.64)	19.70 (0.00, 178.59)
V. cholerae O1	1.02 (0.28, 2.89)	0.65 (0.25, 1.19)	1.81 (1.26, 2.57)
Aeromonas	0.37 (014, 0.82)	0.17 (0.01, 0.53)	0.30 (0.07, 0.67)
E. histolytica	0.68 (0.00, 10.82)	– –	– –

Estimates represent the age-specific clinically-attended, antibiotic-treated MSD adjusted attributable fraction based on qPCR data, divided by the estimate of the same quantity based conventional diagnostic data. Estimates are based on pooled data across all settings, with each setting weighted equally. We present estimates as median (95% CI). We present setting-specific estimates in **S1 Figure**.

S5 Table: Adjusted attributable fractions of clinically-attended, antibiotic-treated moderate-to-severe diarrhea associated with individual pathogens, by age stratum and site, estimated from quantitative molecular diagnostic data.

Pathogen	Setting							
	The Gambia	Mali	Mozambique	Kenya	India	Bangladesh	Pakistan	All (excl. Kenya)[1]
Ages 6 weeks to 11 months								
Rotavirus	29.7 (23.8, 36.4)	34.9 (28.8, 40.9)	54.4 (46.7, 61.3)	22.9 (17.6, 28.7)	42.7 (36.7, 48.7)	35.6 (29.8, 41.4)	17.2 (8.2, 28.2)	38.0 (34.0, 41.5)
Adenovirus 40/41	14.3 (9.5, 19.6)	23.7 (17.3, 29.8)	23.0 (16.8, 29.7)	12.9 (8.7, 17.4)	40.9 (31.5, 48.6)	15.1 (10.9, 19.7)	11.5 (4.6, 19.7)	23.6 (18.1, 28.3)
Shigella	14.2 (9.5, 19.4)	5.7 (3.1, 8.9)	5.4 (2.5, 9.2)	10.9 (7.1, 15.3)	8.3 (5.1, 11.9)	14.3 (10.4, 18.3)	46.2 (34.1, 59.5)	11.2 (9.1, 13.6)
Cryptosporidium	16.9 (11.7, 22.6)	19.7 (14.2, 26.8)	17.7 (11.9, 24.7)	13.6 (9.0, 18.8)	6.3 (3.5, 9.8)	0.9 (0.1, 2.1)	3.3 (0.0, 9.8)	10.9 (8.5, 14.5)
C. jejuni	8.2 (3.4, 13.7)	7.9 (3.5, 12.7)	14.1 (7.6, 21.6)	10.3 (5.2, 16.1)	11.7 (6.7, 17.3)	8.7 (4.1, 14.0)	14.1 (5.7, 24.9)	10.2 (5.3, 15.3)
ST-ETEC (ST-only or LT/ST)	9.3 (5.4, 13.8)	9.9 (5.5, 15.6)	13.9 (8.1, 20.8)	12.3 (7.7, 17.3)	6.8 (3.1, 11.3)	2.2 (0.9, 4.0)	2.6 (0.0, 7.9)	7.6 (4.9, 10.8)
Norovirus GII	4.9 (2.3, 8.3)	6.4 (3.5, 9.8)	5.5 (2.5, 9.0)	4.4 (2.1, 7.6)	5.5 (3.2, 8.6)	2.5 (1.0, 4.6)	4.4 (0.6, 10.7)	4.9 (3.2, 6.9)
Sapovirus	3.8 (1.0, 7.8)	4.9 (2.1, 8.6)	1.6 (0.1, 4.6)	3.2 (0.9, 6.5)	4.0 (1.4, .3)	2.0 (0.5, 4.1)	7.8 (1.6, 16.5)	3.6 (1.4, 6.2)
Astrovirus	2.0 (0.3, 4.7)	3.6 (1.3, 7.2)	0.6 (0.0, 2.6)	2.6 (0.8, 5.2)	4.8 (1.8, 8.7)	2.3 (0.7, 4.8)	2.8 (0.0, 9.0)	2.9 (1.2, 5.5)
tEPEC	1.1 (0.0, 4.7)	2.3 (0.1, 5.9)	2.1 (0.0, 6.1)	1.9 (0.0, 6.6)	1.2 (0.0, 3.7)	0.2 (0.0, 1.6)	0.8 (0.0, 5.7)	1.3 (0.1, 4.0)
Aeromonas	0.1 (0.0, 0.8)	0.2 (0.0, 1.0)	1.7 (0.2, 4.2)	0.5 (0.0, 2.0)	2.1 (0.6, 4.4)	1.1 (0.2, 2.8)	1.5 (1.0, 7.0)	1.1 (0.4, 2.4)
Non-typhoidal *Salmonella*	0.7 (0.0, 2.9)	1.0 (0.0, 2.5)	2.0 (0.2, 5.0)	0.7 (0.0, 2.6)	0.0 (0.0, 0.3)	0.3 (0.0, 1.7)	0.0 (0.0, 0.9)	0.7 (0.1, 1.8)
Vibrio cholerae O1	– –	0.3 (0.0, 1.1)	– –	1.1 (0.0, 2.9)	1.5 (0.3, 3.3)	0.8 (0.0, 2.0)	– –	0.6 (0.2, 1.2)
Entamoeba histolytica	– –	0.1 (0.0, 1.1)	0.0 (0.0, 0.3)	0.2 (0.0, 1.0)	0.0 (0.0, 0.3)	0.6 (0.0, 1.7)	– –	0.2 (0.0, 0.6)
Ages 12 to 23 months								
Shigella	44.4 (37.9, 50.9)	37.8 (32.2, 43.8)	48.0 (38.6, 56.6)	24.1 (18.9, 30.2)	27.1 (21.8, 31.9)	66.3 (60.6, 71.5)	69.9 (59.6, 78.9)	45.8 (42.4, 48.9)
Rotavirus	17.1 (12.5, 22.1)	20.0 (15.3, 24.6)	26.2 (18.8, 34.4)	22.0 (16.4, 27.6)	37.8 (32.2, 43.7)	45.1 (37.6, 51.8)	5.9 (1.9, 11.0)	29.5 (25.7, 33.1)
Adenovirus 40/41	14.5 (9.6, 19.9)	17.9 (11.9, 23.8)	24.3 (16.2, 32.6)	5.7 (3.1, 8.9)	20.6 (14.1, 26.5)	10.7 (6.7, 14.9)	8.9 (3.8, 15.1)	16.4 (11.6, 20.8)
ST-ETEC (ST-only or LT/ST)	11.1 (7.2, 15.7)	9.6 (6.0, 14.6)	19.3 (12.0, 28.2)	9.7 (5.8, 14.4)	8.8 (5.6, 12.9)	3.0 (1.3, 5.1)	8.2 (2.8, 15.3)	8.9 (6.7, 12.1)
Cryptosporidium	6.7 (3.4, 10.8)	6.2 (3.1, 10.2)	10.8 (5.8, 17.2)	5.6 (2.6, 9.5)	5.8 (3.4, 8.7)	1.2 (0.2, 2.6)	5.9 (1.5, 12.4)	5.5 (3.6, 7.9)
C. jejuni	4.2 (0.3, 10.1)	6.0 (0.2, 14.0)	4.2 (0.3, 9.9)	3.2 (0.2, 8.5)	8.6 (0.5, 17.0)	2.4 (0.1, 6.1)	7.1 (0.2, 16.6)	5.4 (0.3, 11.7)
Astrovirus	2.5 (0.6, 5.1)	4.3 (1.2, 7.9)	1.3 (0.0, 3.7)	3.3 (1.2, 6.0)	4.0 (1.4, 7.0)	2.6 (0.6, 5.3)	6.1 (1.3, 13.0)	3.4 (1.2, 5.7)
Sapovirus	2.5 (0.4, 5.9)	2.9 (0.6, 6.5)	2.1 (0.1, 6.4)	3.7 (0.8, 7.6)	4.2 (0.9, 8.6)	2.6 (0.6, 5.5)	5.5 (0.7, 12.6)	3.2 (0.8, 6.4)
Non-typhoidal *Salmonella*	3.1 (0.9, 6.1)	2.9 (1.0, 5.5)	2.9 (0.2, 6.8)	2.3 (0.5, 4.6)	0.4 (0.0, 1.5)	1.5 (0.3, 3.2)	– –	2.0 (0.8, 3.1)
Norovirus GII	2.0 (0.5, 4.3)	0.7 (0.0, 2.3)	1.3 (0.0, 3.6)	1.6 (0.3, 3.6)	4.3 (1.5, 7.6)	1.9 (0.6, 3.9)	4.3 (0.8, 10.0)	2.4 (1.0, 4.1)
Vibrio cholerae O1	– –	0.2 (0.0, 0.9)	1.0 (0.0, 3.6)	0.8 (0.0, 2.5)	4.0 (1.5, 6.9)	0.5 (0.0, 1.6)	1.4 (0.0, 5.2)	1.3 (0.5, 2.3)
Aeromonas	0.0 (0.0, 0.7)	0.8 (0.0, 2.2)	1.4 (0.0, 5.4)	0.2 (0.0, 1.5)	0.9 (0.0, 3.3)	0.6 (0.0, 2.9)	0.8 (0.0, 5.4)	0.7 (0.0, 2.3)
Entamoeba histolytica	– –	– –	0.6 (0.0, 2.5)	– –	0.5 (0.0, 1.7)	2.3 (0.0, 4.5)	1.4 (0.0, 5.1)	0.8 (0.0, 1.5)
tEPEC	0.4 (0.0, 3.0)	0.1 (0.0, 1.9)	0.0 (0.0, 1.6)	0.0 (0.0, 0.9)	0.0 (0.0, 1.1)	0.0 (0.0, 0.4)	0.0 (0.0, 1.2)	0.1 (0.0, 1.4)
Ages 24 to 59 months								
Shigella	37.6 (30.0, 45.1)	29.7 (24.2, 35.8)	53.7 (43.1, 64.5)	26.3 (20.4, 32.3)	40.9 (35.1, 46.5)	82.2 (78.0, 86.3)	55.8 (45.1, 65.8)	51.0 (47.0, 54.7)
Rotavirus	16.6 (11.1, 23.0)	13.7 (9.0, 18.5)	11.8 (6.4, 18.9)	3.4 (1.3, 6.2)	20.6 (15.6, 25.6)	11.2 (7.5, 15.3)	2.6 (0.7, 5.5)	14.5 (11.2, 17.7)
C. jejuni	5.8 (0.6, 12.5)	11.3 (2.9, 19.6)	3.4 (0.6, 8.1)	2.4 (0.3, 5.4)	11.0 (2.9, 18.4)	1.4 (0.2, 2.9)	12.9 (2.0, 24.3)	7.5 (1.7, 13.0)
Adenovirus 40/41	4.3 (0.2, 9.0)	2.3 (0.2, 4.7)	3.9 (0.3, 9.1)	1.9 (0.1, 4.0)	7.5 (0.8, 13.5)	9.9 (0.6, 18.1)	3.4 (0.0, 8.2)	6.2 (0.5, 10.9)
Sapovirus	5.0 (2.1, 8.8)	4.2 (2.1, 6.8)	6.9 (2.5, 12.5)	5.4 (2.7, 8.6)	.8 (4.8, 10.9)	6.1 (3.3, 9.2)	6.1 (2.2, 11.5)	6.1 (3.8, 8.2)
Vibrio cholerae O1	1.2 (0.0, 3.4)	0.7 (0.0, 1.9)	10.6 (4.0, 18.1)	0.9 (0.0, 2.5)	11.9 (8.4, 15.8)	2.6 (1.1, 4.7)	12.5 (5.6, 21.4)	5.7 (4.2, 7.1)
ST-ETEC (ST-only or LT/ST)	6.4 (2.5, 11.2)	3.8 (1.6, 6.6)	8.9 (3.3, 15.8)	6.6 (3.5, 10.4)	5.8 (3.2, 9.3)	1.0 (0.2, 2.4)	2.8 (0.3, 7.3)	4.2 (2.4, 6.3)
Astrovirus	3.8 (0.5, 7.8)	2.4 (0.5, 4.9)	4.0 (0.4, 9.1)	1.2 (0.2, 3.0)	2.7 (1.0, 4.8)	3.5 (0.9, 6.2)	7.1 (1.4, 14.8)	3.4 (1.1, 5.5)
Norovirus GII	1.4 (0.0, 3.6)	2.5 (0.8, 5.0)	0.1 (0.0, 1.6)	2.3 (0.7, 4.5)	3.9 (1.7, 6.8)	2.9 (1.1, 5.4)	3.9 (0.7, 8.8)	2.9 (1.4, 4.7)
Non-typhoidal *Salmonella*	4.4 (1.5, 8.6)	3.1 (1.3, 5.5)	1.9 (0.0, 5.6)	3.7 (1.4, 6.5)	1.2 (0.3, 2.6)	0.6 (0.0, 1.6)	4.3 (0.0, 10.3)	2.1 (1.3, 3.1)
Entamoeba histolytica	– –	2.9 (1.0, 5.5)	– –	0.5 (0.0, 1.5)	2.3 (0.8, 4.2)	0.3 (0.0, 0.9)	9.8 (3.4, 17.6)	1.9 (1.1, 2.9)
Aeromonas	0.0 (0.0, 0.2)	0.3 (0.0, 1.5)	2.8 (0.0, 7.5)	1.3 (0.0, 3.3)	3.2 (0.8, 6.5)	1.6 (0.2, 3.8)	0.3 (0.0, 2.9)	1.6 (0.4, 3.4)
Cryptosporidium	1.2 (0.0, 3.4)	1.1 (0.0, 3.5)	2.6 (0.0, 7.0)	1.6 (0.3, 3.8)	2.8 (1.1, 5.2)	0.1 (0.0, 0.6)	0.9 (0.0, 4.1)	1.5 (0.5, 2.9)
tEPEC	1.0 (0.0, 4.3)	0.7 (0.0, 4.0)	1.0 (0.0, 4.6)	0.3 (0.0, 2.8)	0.6 (0.0, 3.1)	0.2 (0.0, 1.1)	0.9 (0.0, 5.4)	0.7 (0.0, 2.9)
atEPEC	– –	0.0 (0.0, 0.1)	– –	0.0 (0.0, 0.1)	0.2 (0.0, 1.4)	– –	– –	0.1 (0.0, 0.4)

Abbreviations: ST-ETEC (ST-only or LT/ST): Toxigenic *E. coli* encoding Shiga toxin (stable toxin or heat-labile toxin); tEPEC: Typical enteropathogenic *E. coli*; atEPEC: Atypical enteropathogenic *E. coli*. Estimates are presented as adjusted attributable fractions (%) for each pathogen, controlling for quantity of the other pathogens, together with 95% confidence intervals. Empty cells indicate where point estimates of the model-estimated adjusted attributable fraction were zero or below. Estimates exclude diarrhea cases receiving antibiotics who were also diagnosed with pneumonia/lower respiratory tract infection, meningitis or other invasive disease, or typhoid, as these conditions warrant antibiotic treatment irrespective of diarrhea symptoms.

1. Aggregated estimates across sites weight individual cases so that each site receives equal weight within each age stratum.

S6 Table: Adjusted attributable fractions of clinically-attended, antibiotic-treated less-severe diarrhea associated with individual pathogens, by age stratum and site, based on multipliers pooled across sites.

Pathogen	Setting						
	The Gambia	Mali	Mozambique	India	Bangladesh	Pakistan	All (excl. Kenya)[1]
Ages 6 weeks to 11 months							
Rotavirus	25.5 (17.1, 35.9)	25.3 (17.1, 35.3)	48.7 (32.3, 66.5)	10.0 (2.0, 20.0)	61.4 (45.7, 78.0)	13.2 (0.0, 34.7)	32.2 (26.5, 38.8)
Sapovirus	35.4 (0.0, 100.0)	36.4 (0.0, 100.0)	16.2 (0.0, 100.0)	38.5 (0.0, 100.0)	11.5 (0.0, 100.0)	73.1 (0.0, 100.0)	33.0 (0.0, 100.0)
Adenovirus 40/41	12.6 (0.0, 31.8)	14.6 (3.1, 37.0)	45.0 (12.0, 100.0)	– –	72.9 (31.6, 100.0)	56.7 (0.0, 100.0)	22.3 (0.0, 49.1)
Norovirus GII	16.6 (6.8, 50.8)	6.3 (1.6, 21.0)	3.7 (0.0, 15.2)	5.6 (0.0, 23.1)	26.8 (10.9, 79.8)	5.7 (0.0, 34.9)	12.3 (5.6, 37.5)
ST-ETEC (ST-only or LT/ST)	12.6 (5.6, 25.5)	4.6 (1.3, 10.6)	17.0 (6.5, 36.4)	1.8 (0.0, 7.3)	8.0 (2.4, 17.8)	30.7 (9.0, 65.9)	9.6 (4.9, 16.9)
Cryptosporidium	10.6 (6.3, 16.4)	6.9 (3.9, 10.9)	14.2 (7.6, 23.0)	2.7 (0.0, 6.8)	4.5 (1.7, 9.5)	10.7 (0.0, 30.0)	7.0 (0.0, 11.4)
V. cholerae O1	– –	– –	– –	– –	2.5 (0.0, 9.8)	2.5 (0.0, 16.5)	0.5 (0.0, 2.0)
Aeromonas	– –	– –	0.3 (0.0, 1.6)	– –	1.8 (0.1, 5.7)	0.4 (0.0, 3.1)	0.4 (0.0, 1.2)
E. histolytica	– –	– –	0.1 (0.0, 7.0)	0.0 (0.0, 3.7)	0.0 (0.0, 4.7)	– –	0.0 (0.0, 2.1)
Shigella	– –	– –	0.0 (0.0, 3.8)	0.0 (0.0, 3.5)	– –	0.0 (0.0, 19.6)	0.0 (0.0, 1.4)
Ages 12 to 23 months							
Rotavirus	16.9 (9.6, 26.7)	18.0 (10.7, 27.0)	33.9 (20.1, 49.5)	16.8 (7.4, 29.5)	81.2 (61.4, 100.0)	22.7 (0.0, 59.7)	29.8 (23.2, 37.3)
Sapovirus	15.6 (0.0, 100.0)	15.9 (0.0, 100.0)	18.1 (0.0, 100.0)	17.6 (0.0, 100.0)	27.9 (0.0, 100.0)	13.6 (0.0, 100.0)	19.4 (0.0, 100.0)
ST-ETEC (ST-only or LT/ST)	14.3 (7.3, 23.9)	10.6 (5.3, 18.2)	18.6 (9.5, 32.5)	11.6 (4.8, 22.4)	6.7 (1.9, 13.9)	6.9 (0.0, 25.5)	12.3 (7.7, 18.9)
Adenovirus 40/41	8.2 (0.0, 26.4)	3.2 (0.0, 13.8)	23.2 (0.0, 90.5)	6.5 (0.0, 26.5)	32.9 (6.4, 86.3)	– –	10.3 (0.0, 29.2)
Shigella	11.1 (3.5, 21.2)	1.2 (0.0, 4.0)	10.5 (2.4, 21.9)	7.7 (0.0, 18.7)	12.2 (2.7, 24.9)	48.5 (0.0, 100.0)	8.8 (5.3, 12.8)
Cryptosporidium	9.5 (4.9, 19.7)	7.4 (3.7, 13.8)	7.9 (0.0, 19.9)	2.9 (0.6, 8.0)	1.4 (0.0, 4.0)	8.3 (0.0, 29.2)	5.4 (0.0, 12.1)
Norovirus GII	4.8 (1.2, 22.5)	1.5 (0.0, 8.4)	3.5 (0.0, 18.9)	7.8 (2.0, 44.3)	6.7 (1.5, 36.3)	9.4 (0.0, 63.1)	4.6 (1.5, 25.9)
E. histolytica	– –	0.0 (0.0, 2.0)	0.8 (0.0, 15.6)	0.2 (0.0, 8.0)	0.9 (0.0, 19.7)	– –	0.4 (0.0, 6.9)
V. cholerae O1	– –	– –	– –	1.4 (0.0, 4.5)	– –	5.7 (0.0, 21.4)	0.3 (0.1, 1.0)
Aeromonas	– –	– –	0.2 (0.0, 1.1)	– –	0.8 ().0, 3.6)	1.0 (0.0, 6.6)	0.2 (0.0, 0.8)
Ages 24 to 59 months							
Rotavirus	10.1 (3.2, 20.4)	5.2 (1.1, 11.0)	43.3 (21.9, 73.3)	14.9 (6.0, 27.7)	42.7 (22.9, 73.4)	– –	16.1 (10.4, 24.1)
Shigella	18.9 (7.6, 32.3)	– –	17.3 (4.1, 36.1)	2.1 (0.0, 6.9)	48.6 (25.1, 79.9)	– –	11.4 (7.3, 16.1)
ST-ETEC (ST-only or LT/ST)	11.0 (5.0, 22.5)	1.8 (0.4, 4.6)	11.8 (4.2, 26.9)	1.1 (0.0, 3.7)	7.4 (1.9, 18.3)	18.9 (3.8, 47.4)	5.7 (2.7, 10.9)
Norovirus GII	4.2 (0.9, 11.7)	3.7 (1.0, 9.6)	1.7 (0.0, 7.9)	4.1 (0.8, 12.2)	14.7 (4.4, 36.6)	– –	4.9 (2.0, 11.0)
Adenovirus 40/41	3.4 (0.0, 22.9)	2.1 (0.0, 15.0)	7.6 (0.0, 49.9)	3.4 (0.0, 22.8)	8.8 (0.0, 49.7)	– –	4.5 (0.0, 16.9)
E. histolytica	– –	0.9 (0.0, 9.5)	0.4 (0.0, 10.1)	0.7 (0.0, 8.4)	1.4 (0.0, 16.5)	– –	0.8 (0.0, 7.4)
V. cholerae O1	– –	– –	– –	2.3 (0.0, 7.4)	– –	12.8 (0.0, 50.9)	0.8 (0.0, 2.1)
Aeromonas	– –	0.0 (0.0, 0.3)	0.6 (0.0, 2.7)	– –	1.3 (0.0, 4.7)	4.3 (0.0, 16.0)	0.3 (0.0, 1.1)

Abbreviations: ST-ETEC (ST-only or LT/ST): Toxigenic *E. coli* encoding Shiga toxin (stable toxin or heat-labile toxin); tEPEC: Typical enteropathogenic *E. coli*; atEPEC: Atypical enteropathogenic *E. coli*. Estimates are presented as adjusted attributable fractions (%) for each pathogen, controlling for presence of the other pathogens, together with 95% confidence intervals. Empty cells indicate where point estimates of the model-estimated adjusted attributable fraction were zero or below. We did not identify associations of either *C. jejuni* or non-typhoidal *Salmonella* with clinically-attended, antibiotic-treated diarrhea in any age group. Estimates exclude diarrhea cases receiving antibiotics who were also diagnosed with pneumonia/lower respiratory tract infection, meningitis or other invasive disease, or typhoid, as these conditions warrant antibiotic treatment irrespective of diarrhea symptoms.

1. Aggregated estimates across sites weight individual cases so that each site receives equal weight within each age stratum.

S7 Table: Adjusted attributable fractions of clinically-attended, antibiotic-treated less-severe diarrhea associated with individual pathogens, by age stratum and site, estimated from conventional diagnostic data.

Pathogen	Setting						
	The Gambia	Mali	Mozambique	India	Bangladesh	Pakistan	All (excl. Kenya)[1]
Ages 6 weeks to 11 months							
Rotavirus	15.3 (10.3, 21.1)	15.1 (10.4, 20.4)	28.9 (19.6, 38.7)	6.0 (1.2, 11.7)	36.5 (27.6, 44.7)	7.9 (0.0, 20.1)	19.1 (16.4, 22.3)
Cryptosporidium	11.1 (7.3, 15.7)	7.2 (4.3, 10.7)	15.1 (8.2, 22.5)	2.9 (0.0, 6.9)	4.8 (2.0, 9.1)	11.0 (0.0, 29.6)	7.8 (0.0, 10.6)
Norovirus GII	6.2 (3.4, 9.6)	2.3 (0.9, 4.3)	1.5 (0.0, 4.0)	2.2 (0.0, 5.1)	9.9 (5.4, 15.2)	2.3 (0.0, 8.2)	4.6 (3.0, 6.3)
Sapovirus	4.4 (1.7, 7.7)	4.3 (1.9, 7.3)	2.1 (0.0, 5.6)	4.8 (1.4, 9.9)	1.4 (0.0, 3.8)	9.3 (1.8, 20.8)	4.0 (1.8, 6.0)
ST-ETEC (ST-only or LT/ST)	4.8 (2.5, 8.0)	1.8 (0.5, 3.7)	6.7 (2.7, 11.5)	0.7 (0.0, 2.7)	3.1 (1.0, 6.1)	11.8 (3.4, 23.1)	3.7 (2.4, 5.3)
Adenovirus 40/41	1.4 (0.0, 3.2)	1.6 (0.4, 3.6)	5.2 (1.1, 10.5)	– –	7.9 (3.6, 12.3)	6.6 (0.0, 21.1)	2.5 (0.0, 4.5)
Aeromonas	– –	– –	0.8 (0.0, 3.1)	– –	5.2 (0.4, 10.2)	1.5 (0.0, 7.0)	1.1 (0.1, 2.0)
Vibrio cholerae O1	– –	– –	– –	– –	2.6 (0.2, 6.4)	3.2 (0.0, 11.1)	0.5 (0.1, 1.3)
Entamoeba histolytica	– –	– –	1.3 (0.0, 4.2)	0.7 (0.0, 3.1)	0.8 (0.0, 2.9)	– –	0.4 (0.0, 1.1)
Shigella	– –	– –	0.0 (0.0, 1.7)	0.0 (0.0, 1.6)	– –	0.0 (0.0, 8.3)	0.0 (00.0, 0.6)
Campylobacter jejuni	– –	– –	– –	– –	– –	– –	– –
Ages 12 to 23 months							
Rotavirus	9.2 (5.3, 14.0)	9.7 (5.9, 14.1)	18.5 (11.3, 26.2)	9.2 (4.1, 15.7)	43.9 (34.8, 53.6)	12.5 (0.0, 31.4)	16.0 (13.3, 19.0)
Cryptosporidium	10.4 (6.8, 15.3)	8.1 (5.0, 12.0)	9.5 (0.0, 17.7)	3.3 (0.8, 7.5)	1.6 (0.0, 4.0)	10.1 (0.0, 26.6)	6.8 (0.0, 9.6)
ST-ETEC (ST-only or LT/ST)	7.6 (4.3, 11.7)	5.7 (3.1, 8.8)	10.0 (5.7, 15.4)	6.3 (2.7, 11.1)	3.6 (1.0, 6.9)	3.9 (0.0, 13.0)	6.6 (4.6, 8.8)
Shigella	4.3 (1.3, 8.1)	0.6 (0.0, 1.5)	4.1 (1.0, 8.4)	3.0 (0.0, 7.1)	4.8 (1.0, 9.6)	18.8 (0.0, 38.2)	3.4 (2.1, 4.8)
Norovirus GII	3.4 (1.3, 6.1)	1.1 (0.3, 2.5)	2.5 (0.5, 5.5)	5.6 (2.5, 9.9)	4.8 (1.9, 8.8)	7.4 (0.0, 19.0)	3.3 (2.1, 4.7)
Sapovirus	2.4 (0.5, 5.4)	2.5 (0.6, 5.0)	2.9 (0.4, 6.6)	2.9 (0.4, 6.7)	4.5 (1.0, 9.1)	3.6 (0.0, 12.7)	3.0 (0.8, 4.8)
Adenovirus 40/41	1.0 (0.0, 2.7)	0.4 (0.0, 1.4)	2.8 (0.0, 9.0)	0.9 (0.0, 2.8)	4.0 (0.8 , 7.9)	– –	1.3 (0.0, 2.6)
Aeromonas	– –	– –	1.2 (0.0, 3.8)	– –	5.3 (0.4, 10.8)	7.4 (0.0, 21.9)	1.2 (0.1, 2.3)
Entamoeba histolytica	– –	0.1 (0.0, 0.8)	1.4 (0.0, 4.8)	0.6 (0.0, 2.7)	1.8 (0.0 5.0)	– –	0.7 (0.0, 1.6)
Vibrio cholerae O1	– –	– –	– –	2.3 (0.0, 5.9)	– –	10.1 (0.0, 26.8)	0.5 (0.1, 1.2)
Campylobacter jejuni	– –	– –	– –	– –	– –	– –	– –
Ages 24 to 59 months							
Rotavirus	4.8 (1.6, 8.8)	2.4 (0.5, 4.9)	20.2 (10.8, 31.3)	6.9 (3.0, 12.0)	20.0 (11.3, 31.1)	– –	7.4 (5.3, 9.9)
ST-ETEC (ST-only or LT/ST)	10.0 (5.6, 15.5)	1.7 (0.4, 3.4)	10.5 (4.3, 18.2)	1.0 (0.0, 2.7)	6.6 (2.1, 13.0)	17.5 (4.7, 36.3)	5.0 (3.2, 7.1)
Shigella	8.0 (3.3, 13.5)	– –	7.4 (1.8, 15.3)	0.9 (0.0, 2.7)	20.5 (10.7, 32.6)	– –	4.7 (3.1, 6.6)
Cryptosporidium	6.5 (3.0, 11.6)	3.5 (1.3, 6.3)	4.7 (0.0, 10.4)	3.6 (0.7, 7.2)	4.7 (0.9, 10.8)	– –	4.4 (2.9, 6.3)
Norovirus GII	2.6 (0.6, 5.6)	2.3 (0.7, 4.4)	1.1 (0.0, 4.0)	2.6 (0.5, 5.7)	9.2 (3.8, 17.0)	– –	3.0 (1.8, 4.5)
Aeromonas	– –	0.2 (0.0, 0.9)	2.1 (0.0, 6.7)	– –	4.7 (0.3, 11.0)	15.4 (0.0, 37.9)	1.1 (0.1, 2.3)
Adenovirus 40/41	0.8 (0.0, 2.4)	05. (0.0, 1.5)	1.6 (0.0, 5.4)	0.7 (0.0, 2.5)	1.6 (0.0, 5.4)	– –	0.8 (0.2, 1.6)
Entamoeba histolytica	– –	0.7 (0.0, 2.3)	0.6 (0.0, 3.1)	0.5 (0.0, 2.5)	1.1 (0.0, 4.8)	– –	0.7 (0.0, 1.6)
Vibrio cholerae O1	– –	– –	– –	1.4 (0.0, 3.7	– –	7.6 (0.0, 26.5)	0.4 (0.0, 1.1)
Sapovirus	0.0 (4.9, 0.2)	0.0 (0.0, 0.5)	– –	0.0 (0.0, 0.7)	0.0 (0.0, 1.7)	– –	0.0 (0.0, 0.6)
Campylobacter jejuni	– –	– –	– –	– –	– –	– –	– –

Abbreviations: ST-ETEC (ST-only or LT/ST): Toxigenic *E. coli* encoding Shiga toxin (stable toxin or heat-labile toxin); tEPEC: Typical enteropathogenic *E. coli*; atEPEC: Atypical enteropathogenic *E. coli*. Estimates are presented as adjusted attributable fractions (%) for each pathogen, controlling for presence of the other pathogens, together with 95% confidence intervals. Empty cells indicate where point estimates of the model-estimated adjusted attributable fraction were zero or below. Estimates exclude diarrhea cases receiving antibiotics who were also diagnosed with pneumonia/lower respiratory tract infection, meningitis or other invasive disease, or typhoid, as these conditions warrant antibiotic treatment irrespective of diarrhea symptoms.

1. Aggregated estimates across sites weight individual cases so that each site receives equal weight within each age stratum.

S8 Table: Adjusted attributable fractions of clinically-attended, antibiotic-treated moderate-to-severe diarrhea associated with individual pathogens, by age stratum and site, estimated from conventional diagnostic data.

Pathogen	Setting							
	The Gambia	Mali	Mozambique	Kenya	India	Bangladesh	Pakistan	All (excl. Kenya)[1]
Age 6 weeks to 11 months								
Rotavirus	24.3 (20.2, 28.9)	19.5 (16.9, 22.0)	40.7 (34.8, 46.7)	18.9 (15.6, 22.4)	25.5 (22.5, 28.5)	16.6 (13.8, 19.4)	11.9 (6.3, 17.2)	22.5 (21.1, 24.0)
Cryptosporidium	13.3 (10.0, 16.7)	13.4 (11.3, 15.6)	15.0 (11.6, 19.0)	13.7 (11.0, 16.4)	12.1 (10.0, 14.2)	5.8 (4.2, 7.5)	9.6 (5.6, 14.5)	11.4 (10.2, 12.6)
Shigella	4.6 (2.6, 6.9)	0.5 (0.1, 1.0)	1.1 (0.0, 2.5)	5.1 (3.0, 7.4)	1.8 (0.9, 2.7)	13.6 (11.0, 16.3)	19.7 (13.8, 26.3)	5.0 (4.2, 5.8)
C. jejuni	0.9 (0.3, 1.6)	0.6 (0.3, 1.1)	0.7 (0.1, 1.4)	3.4 (2.0, 4.9)	3.8 (2.2, 5.4)	7.7 (5.3, 10.1)	6.6 (3.7, 9.8)	3.3 (2.2, 4.3)
Aeromonas	– –	– –	0.6 (0.0, 1.3)	– –	0.3 (0.1, 0.7)	11.5 (8.5, 14.3)	10.3 (0.0, 16.9)	3.1 (2.0, 3.8)
ST-ETEC (ST-only or LT/ST)	5.8 (3.7, 8.0)	3.8 (2.8, 5.1)	3.4 (1.7, 5.3)	5.9 (4.1, 7.8)	2.4 (1.5, 3.4)	1.2 (0.2, 2.1)	0.7 (0.0, 4.0)	2.9 (2.1, 3.6)
Adenovirus 40/41	3.0 (1.5, 5.0)	1.8 (1.1, 2.8)	2.3 (0.7, 4.0)	2.7 (1.5, 4.1)	3.7 (2.5, 4.9)	2.6 (0.0, 4.2)	0.6 (0.0, 2.0)	2.6 (1.4, 3.3)
Norovirus GII	5.5 (2.6, 7.9)	0.5 (0.0, 1.1)	2.0 (1.0, 3.4)	2.4 (0.0, 4.0)	2.1 (0.0, 3.8)	0.9 (0.4, 1.5)	3.0 (0.0, 6.2)	1.8 (0.5, 2.7)
Vibrio cholerae O1	– –	– –	– –	0.1 (0.0, 0.5)	0.8 (03, 1.6)	1.5 (0.6, 2.7)	0.8 (0.0, 2.3)	0.6 (0.2, 0.9)
Non-typhoidal *Salmonella*	0.4 (0.0, 1.2)	– –	0.8 (0.2, 1.8)	0.0 (0.0, 3.7)	0.1 (0.0, 0.4)	0.9 (0.0, 3.5)	1.9 (0.4, 4.3)	0.4 (0.0, 1.1)
Sapovirus	0.3 (0.0, 1.0)	0.5 (0.0, 1.2)	0.2 (0.0, 0.7)	0.2 (0.0, 0.6)	0.4 (0.0, 1.0)	0.1 (0.0, 0.3)	1.0 (0.0, 2.9)	0.4 (0.0, 0.9)
Entamoeba histolytica	0.2 (0.0, 0.6)	0.3 (0.0, 0.7)	0.0 (0.0, 3.6)	0.0 (0.0, 0.5)	0.5 (0.0, 1.2)	0.4 (0.0, 3.3)	0.9 (0.0, 2.4)	0.2 (0.0, 1.3)
Age 12 to 23 months								
Shigella	14.7 (11.7, 18.1)	2.9 (1.8, 4.2)	5.9 (2.6, 10.0)	7.8 (5.0, 10.9)	5.9 (4.3, 7.7)	55.5 (51.3, 59.3)	29.7 (22.0, 36.8)	17.5 (16.1, 18.9)
Rotavirus	13.2 (10.1, 16.2)	13.4 (11.1, 15.8)	21.9 (15.8, 28.7)	14.8 (11.0, 18.9)	23.1 (20.1, 26.2)	13.5 (10.8, 16.2)	6.5 (2.6, 10.4)	15.9 (14.7, 17.1)
Cryptosporidium	8.1 (5.6 10.5)	6.2 (4.6, 7.8)	7.9 (4.3, 12.4)	5.7 (3.5, 7.9)	8.2 (5.5, 10.5)	1.5 (0.0, 4.2)	3.3 (0.0, 6.5)	6.1 (4.0, 7.5)
ST-ETEC (ST-only or LT/ST)	9.3 (7.2, 12.0)	3.9 (2.9, 5.1)	10.3 (6.1, 14.4)	8.1 (5.6, 10.9)	5.0 (3.7, 6.4)	1.0 (0.0, 2.4)	4.1 (0.0, 7.6)	4.8 (3.8, 5.7)
Aeromonas	– –	0.2 (0.0, 0.5)	0.7 (0.0, 1.7)	– –	0.1 (0.0, 0.4)	18.2 (13.8, 21.6)	15.8 (10.6, 21.3)	4.5 (3.5, 5.3)
C. jejuni	0.8 (0.3, 1.4)	0.4 (0.1, 0.7)	– –	3.0 (1.7, 4.5)	4.0 (2.3, 5.6)	4.4 (3.0, 5.8)	9.9 (6.5, 13.6)	2.7 (1.9, 3.5)
Adenovirus 40/41	1.4 (0.6, 2.5)	1.7 (0.9, 2.6)	1.2 (0.0, 3.1)	2.0 (0.6, 3.5)	3.6 (0.6, 5.1)	1.1 (0.0, 2.2)	1.2 (0.0, 3.1)	2.0 (0.7, 2.5)
Vibrio cholerae O1	– –	– –	1.8 (0.0, 4.0)	0.9 (0.0, 2.0)	4.4 (3.0, 6.1)	3.2 (1.8, 4.6)	3.8 (1.2, 7.1)	2.0 (1.6, 2.6)
Norovirus GII	3.2 (0.4, 5.1)	0.0 (0.0, 0.8)	0.6 (0.0, 1.6)	0.0 (0.0, 1.3)	2.8 (0.4, 4.3)	1.4 (0.0, 2.4)	4.1 (2.1, 6.7)	1.7 (0.2, 2.6)
Entamoeba histolytica	0.1 (0.0, 0.5)	0.2 (0.0, 0.7)	4.7 (0.0, 9.4)	0.2 (0.0, 1.2)	0.0 (0.0, 0.7)	2.6 (0.0, 4.9)	0.4 (0.0, 1.4)	0.6 (0.0, 1.5)
Sapovirus	0.5 (0.0, 1.4)	0.3 (0.0, 0.9)	0.0 (0.0, 0.4)	0.6 (0.0, 1.7)	0.5 (0.0, 1.4)	0.3 (0.0, 0.9)	0.7 (0.0, 2.1)	0.4 (0.0, 1.0)
Non-typhoidal *Salmonella*	0.8 (0.2, 1.7)	0.1 (0.0, 0.3)	– –	4.8 (2.9, 7.5)	0.2 (0.0, 0.6)	0.0 (0.0, 1.4)	0.0 (0.0, 3.2)	0.0 (0.0, 0.5)
Age 24 to 59 months								
Shigella	12.4 (7.3, 17.6)	2.3 (1.3, 3.5)	18.8 (11.7, 26.8)	11.6 (8.3, 15.3)	9.3 (6.8, 12.0)	68.8 (64.0, 73.0)	26.1 (16.6, 35.1)	21.3 (19.5, 23.1)
Rotavirus	10.5 (6.9, 14.6)	3.6 (2.5, 4.9)	8.2 (3.6, 13.6)	2.9 (1.3, 4.9)	10.9 (8.2, 13.7)	6.5 (4.3, 8.8)	1.9 (0.0, 4.7)	6.7 (5.6, 7.8)
Aeromonas	– –	0.1 (0.0, 0.4)	0.5 (0.0, 1.7)	0.3 (0.0, 1.0)	0.2 (0.0, 0.6)	21.3 (17.3, 25.1)	14.4 (0.0, 23.2)	5.4 (4.1, 6.5)
ST-ETEC (ST-only or LT/ST)	6.6 (3.7, 9.8)	3.2 (2.2, 4.3)	6.8 (2.9, 11.4)	5.4 (3.4, 7.9)	4.9 (3.4, 6.7)	2.5 (1.4, 3.9)	0.0 (0.0, 5.9)	3.8 (2.6, 4.7)
Vibrio cholerae O1	– –	– –	6.8 (1.9, 12.6)	0.3 (0.0, 1.3)	6.7 (4.6, 9.1)	3.9 (2.2, 5.7)	8.4 (3.7, 14.2)	3.2 (2.4, 4.0)
C. jejuni	0.4 (0.0, 1.1)	0.6 (0.3, 1.0)	– –	2.1 (1.2, 3.3)	4.0 (2.5, 5.6)	1.2 (0.6, 2.0)	9.9 (6.4, 14.2)	2.0 (1.3, 2.6)
Norovirus GII	3.0 (0.0, 5.5)	1.0 (0.5, 1.5)	0.5 (0.0, 1.7)	1.1 (0.4, 1.9)	1.4 (0.7, 2.2)	2.5 (1.5, 3.7)	4.5 (2.0, 7.7)	1.8 (1.1, 2.4)
Entamoeba histolytica	– –	1.1 (0.3, 1.8)	0.6 (0.0, 6.9)	0.6 (0.0, 1.4)	1.7 (0.8, 3.0)	1.9 (0.0, 4.0)	3.6 (1.2, 6.9)	1.3 (0.0, 2.2)
Sapovirus	0.7 (0.0, 2.1)	0.2 (0.0, 0.6)	– –	0.2 (0.0, 0.8)	0.3 (0.0, 1.0)	0.1 (0.0, 0.3)	0.4 (0.0, 1.6)	0.3 (0.0, 0.7)
Adenovirus 40/41	0.9 (0.0, 2.3)	0.7 (0.2, 1.4)	– –	1.2 (0.3, 2.4)	2.3 (1.1, 3.5)	0.4 (0.0, 1.3)	– –	0.9 (0.0, 1.4)
Non-typhoidal *Salmonella*	0.0 (0.0, 3.1)	0.1 (0.0, 0.4)	– –	4.8 (2.7, 7.4)	0.5 (0.0, 1.2)	0.0 (0.0, 1.9)	0.0 (0.0, 3.5)	0.0 (0.0, 0.8)
Cryptosporidium	0.5 (0.0, 2.0)	1.0 (0.0, 2.0)	0.8 (0.0, 3.5)	1.0 (0.0, 2.5)	1.9 (0.0, 4.3)	0.0 (0.0, 0.1)	0.0 (0.0, 0.8)	0.0 (0.0, 1.6)

Abbreviations: ST-ETEC (ST-only or LT/ST): Toxigenic *E. coli* encoding Shiga toxin (stable toxin or heat-labile toxin); tEPEC: Typical enteropathogenic *E. coli*; atEPEC: Atypical enteropathogenic *E. coli*. Estimates are presented as adjusted attributable fractions (%) for each pathogen, controlling for presence of the other pathogens. Models are fitted with data from all MSD cases as well as their matched controls. Empty cells indicate where point estimates of the model-estimated adjusted attributable fraction were zero or below. Estimates exclude diarrhea cases receiving antibiotics who were also diagnosed with pneumonia/lower respiratory tract infection, meningitis or other invasive disease, or typhoid, as these conditions warrant antibiotic treatment irrespective of diarrhea symptoms.

1. Aggregated estimates across sites weight individual cases so that each site receives equal weight within each age stratum.

S9 Table: Adjusted attributable fractions of all clinically-attended, antibiotic-treated diarrhea associated with individual pathogens, by age stratum and site, estimated from conventional diagnostic data for both moderate-to-severe diarrhea and less-severe diarrhea.

Pathogen	Setting						
	The Gambia	Mali	Mozambique	India	Bangladesh	Pakistan	All (excl. Kenya)[1]
Age 6 weeks to 11 months							
Rotavirus	17.0 (12.5, 21.8)	17.9 (14.4, 20.7)	33.3 (23.6, 40.8)	17.7 (12.8, 21.8)	26.1 (17.4, 36.3)	8.7 (2.3, 18.0)	20.6 (17.4, 25.5)
Cryptosporidium	11.5 (8.1, 15.4)	11.0 (7.9, 13.9)	15.1 (9.8, 20.5)	8.4 (5.9, 11.0)	5.4 (3.2, 7.8)	10.8 (1.3, 24.4)	9.6 (0.0, 12.9)
Shigella	2.5 (0.9, 5.5)	3.4 (1.2, 6.7)	1.8 (0.4, 5.5)	5.0 (2.7, 8.1)	7.2 (1.7, 14.2)	13.5 (5.9, 29.6)	4.2 (2.5, 6.3)
ST-ETEC (ST-only or LT/ST)	5.0 (3.1, 7.7)	3.1 (1.9, 4.3)	5.4 (2.9, 9.7)	1.7 (0.9, 2.7)	2.0 (0.8, 4.2)	8.8 (2.7, 17.3)	3.9 (2.8, 5.8)
Norovirus GII	6.1 (3.6, 9.0)	1.2 (0.4, 2.5)	1.6 (0.3, 3.5)	2.0 (0.5, 3.7)	5.1 (1.4, 10.9)	2.3 (0.0, 6.9)	3.3 (1.9, 5.4)
Sapovirus	3.6 (1.4, 6.5)	1.9 (0.6, 4.4)	1.3 (0.0, 3.8)	2.2 (0.8, 4.8)	0.6 (0.0, 2.4)	6.9 (1.6, 15.7)	2.6 (1.2, 4.3)
Adenovirus 40/41	1.7 (0.5, 3.3)	1.8 (0.9, 2.7)	4.0 (1.4, 8.1)	2.2 (1.3, 3.3)	5.1 (2.3, 9.6)	4.4 (0.0, 16.3)	2.5 (0.0, 4.7)
Aeromonas	– –	– –	0.8 (0.1, 2.4)	0.2 (0.0, 0.4)	8.5 (4.1, 12.3)	3.8 (0.4, 8.6)	1.4 (0.7, 2.7)
Campylobacter jejuni	0.2 (0.0, 0.4)	0.4 (0.1, 0.8)	0.2 (0.0, 0.8)	2.2 (1.2, 3.6)	3.8 (0.9, 7.8)	1.7 (0.7, 3.7)	1.2 (0.6, 1.9)
Vibrio cholerae O1	– –	– –	– –	0.5 (0.2, 0.9)	2.0 (0.7, 4.5)	2.4 (0.0, 8.7)	0.5 (0.2, 1.3)
Entamoeba histolytica	0.0 (0.0, 0.1)	0.1 (0.0, 0.5)	1.0 (0.0, 3.3)	0.6 (0.1, 1.7)	0.8 (0.0, 2.7)	0.2 (0.0, 0.8)	0.5 (0.1, 1.3)
Non-typhoidal *Salmonella*	0.1 (0.0, 0.3)	– –	0.2 (0.0, 0.9)	0.1 (0.0, 0.2)	0.3 (0.0, 2.5)	0.5 (0.1, 1.4)	0.2 (0.0, 0.4)
Age 12 to 23 months							
Shigella	19.5 (11.6, 29.0)	22.0 (7.9, 36.6)	20.9 (10.5, 38.1)	19.5 (12.6, 25.9)	37.3 (19.7, 57.9)	55.4 (21.2, 90.3)	23.5 (17.6, 29.4)
Rotavirus	10.2 (7.0, 14.0)	11.9 (8.7, 14.6)	19.5 (13.9, 25.5)	17.8 (13.2, 21.9)	29.5 (18.3, 41.7)	10.5 (1.1, 23.4)	16.3 (13.4, 20.2)
Cryptosporidium	9.8 (7.1, 13.7)	7.0 (5.3, 9.2)	8.9 (0.8, 14.9)	6.3 (4.0, 8.8)	1.5 (0.0, 3.5)	7.7 (0.4, 19.2)	6.5 (0.0, 9.7)
ST-ETEC (ST-only or LT/ST)	8.1 (5.3, 11.3)	4.6 (3.3, 6.8)	10.1 (6.6, 14.2)	5.4 (3.8, 7.7)	2.3 (0.7, 4.8)	3.8 (0.3, 10.6)	6.5 (4.9, 8.4)
Norovirus GII	3.3 (1.6, 5.5)	0.6 (0.0, 1.6)	1.9 (0.4, 4.4)	3.8 (2.0, 6.1)	3.1 (1.3, 5.9)	6.3 (0.7, 15.2)	3.1 (2.0, 4.3)
Aeromonas	– –	0.1 (0.0, 0.3)	1.1 (0.1, 3.0)	0.1 (0.0, 0.3)	11.4 (5.7, 16.7)	10.1 (3.8, 20.3)	2.0 (0.9, 3.6)
Sapovirus	2.0 (0.5, 4.3)	1.2 (0.2, 3.0)	2.0 (0.3, 5.0)	1.4 (0.4, 3.4)	2.4 (0.5, 5.8)	2.5 (0.0, 9.4)	2.0 (0.7, 3.4)
Adenovirus 40/41	1.1 (0.2, 2.5)	1.1 (0.3, 2.0)	2.2 (0.0, 6.9)	2.5 (1.2, 3.8)	2.6 (0.9, 5.2)	0.3 (0.0, 1.2)	1.6 (0.0, 3.2)
Vibrio cholerae O1	– –	– –	0.4 (0.0, 1.6)	3.6 (2.2, 5.2)	1.4 (0.4, 2.9)	7.8 (0.7, 19.9)	1.6 (0.9, 3.0)
Campylobacter jejuni	0.2 (0.1, 0.5)	0.2 (0.0, 0.5)	– –	2.4 (1.2, 3.8)	2.0 (0.7, 3.8)	2.8 (1.1, 6.4)	1.1 (0.6, 1.7)
Entamoeba histolytica	0.0 (0.0, 0.1)	0.2 (0.0, 0.6)	2.3 (0.3, 5.7)	0.3 (0.0, 1.3)	2.2 (0.2, 4.4)	0.1 (0.0, 0.5)	0.8 (0.2, 1.7)
Non-typhoidal *Salmonella*	0.2 (0.0, 0.5)	0.0 (0.0, 0.2)	– –	0.1 (0.0, 0.4)	0.0 (0.0, 0.8)	0.0 (0.0, 1.0)	0.1 (0.0, 0.2)
Age 24 to 59 months							
Shigella	24.2 (13.7, 34.6)	13.5 (2.2, 28.3)	27.4 (9.5, 49.6)	16.9 (5.0, 30.8)	74.6 (45.3, 84.0)	17.0 (5.5, 35.6)	24.6 (15.3, 34.4)
Rotavirus	6.5 (3.5, 9.8)	3.0 (1.6, 4.6)	16.7 (8.6, 27.2)	8.6 (5.0, 11.9)	9.8 (5.3, 22.7)	0.5 (0.0, 1.9)	8.5 (5.6, 13.5)
ST-ETEC (ST-only or LT/ST)	8.9 (5.7, 13.8)	2.5 (1.2, 3.6)	9.4 (5.0, 15.8)	2.5 (1.0, 4.4)	3.6 (1.9, 8.8)	11.8 (2.5, 26.7)	5.6 (3.7, 9.0)
Aeromonas	– –	0.2 (0.0, 0.6)	1.6 (0.0, 5.5)	0.1 (0.0, 0.3)	16.5 (5.5, 23.3)	14.5 (3.9, 31.4)	2.8 (1.1, 5.8)
Cryptosporidium	4.7 (2.3, 8.8)	2.2 (0.7, 4.6)	3.4 (0.1, 8.4)	3.0 (0.8, 5.6)	1.0 (0.0, 6.2)	0.0 (0.0, 0.2)	2.5 (0.0, 4.6)
Norovirus GII	2.7 (1.0, 5.0)	1.6 (0.8, 3.3)	0.9 (0.0, 3.3)	2.1 (0.9, 4.3)	4.2 (1.9, 11.1)	1.3 (0.3, 3.5)	2.2 (1.2, 3.9)
Vibrio cholerae O1	– –	– –	1.5 (0.2, 6.6)	3.5 (1.2, 5.9)	2.7 (0.4, 5.0)	7.6 (0.9, 20.6)	2.2 (0.9, 4.0)
Entamoeba histolytica	– –	0.9 (0.2, 1.8)	0.8 (0.0, 3.8)	1.1 (0.3, 2.3)	1.7 (0.0, 3.8)	1.1 (0.2, 2.9)	0.9 (0.4, 1.7)
Campylobacter jejuni	0.1 (0.0, 0.4)	0.3 (0.0, 0.7)	– –	1.5 (0.4, 3.2)	0.8 (0.1, 1.7)	3.0 (1.0, 6.6)	0.8 (0.4, 1.7)
Adenovirus 40/41	0.7 (0.0, 2.1)	0.5 (0.1, 1.3)	1.1 (0.0, 4.5)	1.3 (0.3, 2.5)	0.7 (0.0, 3.2)	– –	0.7 (0.0, 1.8)
Sapovirus	0.2 (0.0, 0.8)	0.1 (0.0, 0.5)	– –	0.1 (0.0, 0.6)	0.0 (0.0, 0.4)	0.1 (0.0, 0.6)	0.1 (0.0, 0.4)
Non-typhoidal *Salmonella*	0.0 (0.0, 1.0)	0.0 (0.0, 0.2)	– –	0.2 (0.0, 0.6)	0.0 (0.0, 1.5)	0.0 (0.0, 1.4)	0.1 (0.0, 0.4)

Abbreviations: ST-ETEC (ST-only or LT/ST): Toxigenic *E. coli* encoding Shiga toxin (stable toxin or heat-labile toxin); tEPEC: Typical enteropathogenic *E. coli*; atEPEC: Atypical enteropathogenic *E. coli*. Estimates are presented as adjusted attributable fractions (%) for each pathogen, controlling for presence of the other pathogens, together with 95% confidence interavls.. Empty cells indicate where point estimates of the model-estimated adjusted attributable fraction were zero or below. Estimates exclude diarrhea cases receiving antibiotics who were also diagnosed with pneumonia/lower respiratory tract infection, meningitis or other invasive disease, or typhoid, as these conditions warrant antibiotic treatment irrespective of diarrhea symptoms.

1. Aggregated estimates across sites weight individual cases so that each site receives equal weight within each age stratum

S10 Table: Comparison of adjusted attributable fractions estimates for individual pathogens in clinically-attended, antibiotic-treated moderate-to-severe diarrhea with and without verified antibiotic administration.

Pathogen	Endpoint		
	Clinically-attended, antibiotic-treated diarrhea with verified antibiotic administration	Clinically-attended, antibiotic-treated diarrhea without verified antibiotic administration	All clinically-attended, antibiotic-treated diarrhea
Age 6 weeks to 11 months			
Rotavirus	59.0 (53.0, 64.3)	35.2 (31.0, 39.0)	38.0 (34.0, 41.5)
Adenovirus 40/41	35.8 (24.7, 43.5)	21.3 (14.7, 26.7)	23.6 (18.1, 28.3)
Shigella	5.6 (3.0, 8.7)	13.0 (10.5, 15.5)	11.2 (9.1, 13.6)
Cryptosporidium	12.5 (7.8, 20.3)	11.1 (8.5, 14.7)	10.9 (8.5, 14.5)
C. jejuni	6.4 (0.0, 16.7)	10.3 (4.9, 15.4)	10.2 (5.3, 15.3)
ST-ETEC (ST-only or LT/ST)	11.2 (5.8, 19.1)	7.1 (4.2, 10.2)	7.6 (4.9, 10.8)
Norovirus GII	4.1 (1.4, 7.7)	4.9 (2.8, 7.0)	4.9 (3.2, 6.9)
Sapovirus	4.0 (0.0, 8.7)	3.1 (1.1, 5.9)	3.6 (1.4, 6.2)
Astrovirus	4.8 (0.0, 11.0)	3.3 (1.7, 6.0)	2.9 (1.2, 5.5)
tEPEC	3.3 (0.3, 8.2)	0.5 (0.0, 3.5)	1.3 (0.1, 4.0)
Aeromonas	0.8 (0.0, 2.0)	0.8 (0.2, 1.9)	1.1 (0.4, 2.4)
Non-typhoidal *Salmonella*	1.2 (0.0, 3.6)	0.4 (0.0, 1.2)	0.7 (0.1, 1.8)
Vibrio cholerae O1	0.6 (0.0, 1.8)	0.6 (0.1, 1.2)	0.6 (0.2, 1.2)
Entamoeba histolytica	0.3 (0.0, 1.2)	0.2 (0.0, 0.6)	0.2 (0.0, 0.6)
Age 12 to 23 months			
Shigella	37.7 (31.3, 44.3)	46.8 (43.3, 50.1)	45.8 (42.4, 48.9)
Rotavirus	47.9 (40.6, 54.0)	28.3 (23.8, 32.0)	29.5 (25.7, 33.1)
Adenovirus 40/41	27.6 (16.7, 34.9)	15.9 (10.2, 20.5)	16.4 (11.6, 20.8)
ST-ETEC (ST-only or LT/ST)	14.2 (8.6, 21.1)	8.1 (5.8, 10.8)	8.9 (6.7, 12.1)
Cryptosporidium	6.8 (3.1, 12.1)	5.2 (3.2, 7.6)	5.5 (3.6, 7.9)
C. jejuni	0.3 (0.0, 8.8)	5.2 (0.6, 11.0)	5.4 (0.3, 11.7)
Astrovirus	3.9 (0.0, 8.7)	3.8 (1.5, 6.2)	3.4 (1.2, 5.7)
Sapovirus	1.7 (0.0, 10.4)	3.0 (0.9, 6.4)	3.2 (0.8, 6.4)
Non-typhoidal *Salmonella*	3.2 (0.1, 5.9)	1.9 (0.8, 3.0)	2.0 (0.8, 3.1)
Norovirus GII	5.9 (2.8, 10.1)	2.0 (0.6, 4.0)	2.4 (1.0, 4.1)
Vibrio cholerae O1	2.0 (0.0, 3.9)	1.4 (0.5, 2.4)	1.3 (0.5, 2.3)
Aeromonas	1.3 (0.0, 4.3)	0.8 (0.0, 2.4)	0.7 (0.0, 2.3)
Entamoeba histolytica	0.3 (0.0, 1.8)	0.8 (0.0, 1.5)	0.8 (0.0, 1.5)
tEPEC	0.0 (0.0, 0.8)	0.0 (0.0, 1.6)	0.1 (0.0, 1.4)
Age 24 to 59 months			
Shigella	49.0 (41.1, 57.2)	52.8 (48.9, 56.4)	51.0 (47.0, 54.7)
Rotavirus	18.7 (13.4, 23.7)	14.0 (10.1, 17.4)	14.5 (11.2, 17.7)
C. jejuni	0.5 (0.0, 8.6)	8.1 (2.2, 13.5)	7.5 (1.7, 13.0)
Adenovirus 40/41	11.4 (0.0, 21.1)	6.2 (0.2, 11.3)	6.2 (0.5, 10.9)
Sapovirus	4.7 (0.1, 8.4)	5.8 (3.2, 8.1)	6.1 (3.8, 8.2)
Vibrio cholerae O1	9.0 (4.5, 12.7)	5.8 (4.4, 7.5)	5.7 (4.2, 7.1)
ST-ETEC (ST-only or LT/ST)	9.4 (4.1, 17.2)	4.1 (2.3, 6.1)	4.2 (2.4, 6.3)
Astrovirus	5.3 (0.5, 8.9)	2.9 (0.8, 5.2)	3.4 (1.1, 5.5)
Norovirus GII	0.4 (0.0, 2.3)	3.0 (1.4, 5.0)	2.9 (1.4, 4.7)
Non-typhoidal *Salmonella*	3.0 (1.1, 5.2)	1.9 (1.0, 2.9)	2.1 (1.3, 3.1)
Entamoeba histolytica	0.0 (0.0, 1.1)	2.0 (1.2, 3.0)	1.9 (1.1, 2.9)
Aeromonas	3.4 (1.0, 6.2)	1.5 (0.4, 3.2)	1.6 (0.4, 3.4)
Cryptosporidium	2.9 (0.5, 7.1)	1.4 (0.5, 3.1)	1.5 (0.5, 2.9)
tEPEC	0.1 (0.0, 2.6)	0.4 (0.0, 3.0)	0.7 (0.0, 2.9)
atEPEC	0.0 (0.0, 0.8)	0.1 (0.0, 0.4)	0.1 (0.0, 0.4)

Abbreviations: ST-ETEC (ST-only or LT/ST): Toxigenic *E. coli* encoding Shiga toxin (stable toxin or heat-labile toxin); tEPEC: Typical enteropathogenic *E. coli*; atEPEC: Atypical enteropathogenic *E. coli*.

Estimates are presented as adjusted attributable fractions (%) for each pathogen, controlling for quantity of the other pathogens, together with 95% confidence intervals. Estimates are aggregated across sites and weight individual cases so that each site receives equal weight within each age stratum. Estimates exclude diarrhea cases receiving antibiotics who were also diagnosed with pneumonia/lower respiratory tract infection, meningitis or other invasive disease, or typhoid, as these conditions warrant antibiotic treatment irrespective of diarrhea symptoms.

S11 Table: Data sources.

Estimand	Source	Approach
Incidence rate of diarrhea and proportion of cases treated at study clinics	Healthcare attitudes and utilization survey (HUAS) and HUAS-lite surveys	The diarrhea incidence rate in the population of each Demographic Surveillance Site population is estimated from the proportion of children who experienced acute, new-onset diarrhea episodes in a 7-day recall period before the HUAS/HUAS-lite interview as detailed by Blackwelder and colleagues[3], and the proportion of these children who received care at sentinel clinics
Adjusted attributable fraction in moderate-to-severe diarrhea	TaqMan array card re-analyses of specimens from 300 moderate-to-severe diarrhea cases within each site and age stratum, and first available matched controls[4]	We fitted conditional logistic regression models to estimate the adjusted association of case status with abundance (Cq value) of each pathogen
Uncorrected adjusted attributable fraction in moderate-to-severe diarrhea (conventional diagnostic data)	Qualitative detection of each pathogen among moderate-to-severe diarrhea cases and matched controls based on conventional diagnostic assays	We fitted conditional logistic regression models to estimate the adjusted association of case status with detection (binary) of each pathogen or pairwise combination of pathogens based on stepwise model selection
Uncorrected adjusted attributable fraction in less-severe diarrhea (conventional diagnostic data)	Qualitative detection of each pathogen among less-severe diarrhea cases and matched controls based on conventional diagnostic assays	We fitted conditional logistic regression models to estimate the adjusted association of case status with detection (binary) of each pathogen or pairwise combination of pathogens based on stepwise model selection
Corrected adjusted pathogen-attributable fraction in less-severe diarrhea		We multiplied estimates of the uncorrected adjusted attributable fraction in less-severe diarrhea by the ratio of estimates of the corrected-to-uncorrected adjusted attributable fraction in moderate-to-severe diarrhea

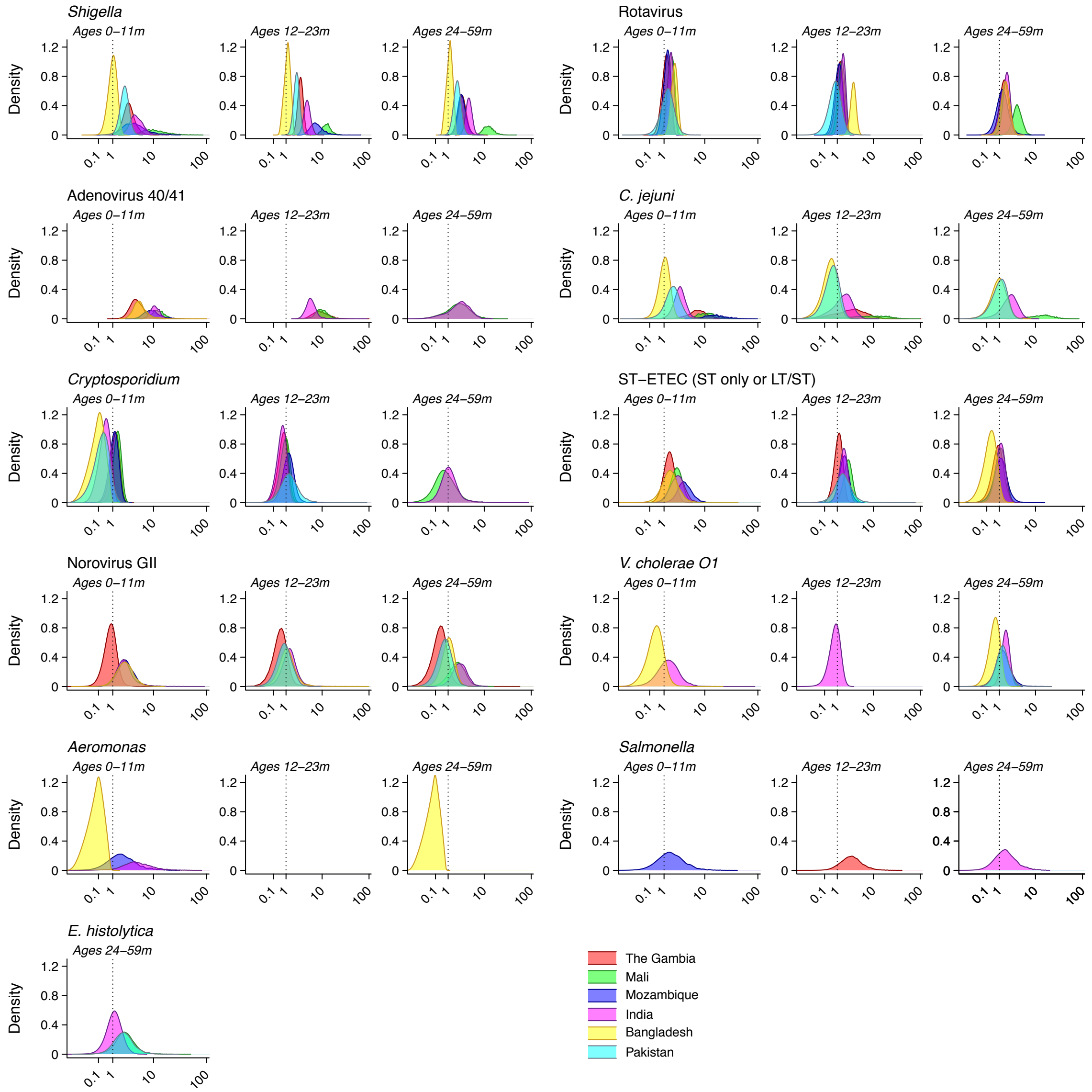

S1 Figure. Multipliers for etiologic fractions based on site-specific estimates. We illustrate estimated multipliers for the relative proportion of clinically-attended, antibiotic-treated MSD attributed to each pathogen, by age and setting, on the basis of quantitative diagnostics (relative to conventional diagnostics). We use the same multipliers to estimate the fraction of clinically-attended, antibiotic-treated LSD attributable to each pathogen, on the basis of conventional microbiological data obtained in the primary study. The x-axis is plotted on the scale of *ln*(1+x) to conserve *x* values equal to zero.

Abbreviations: ST-ETEC (ST-only or LT/ST): Toxigenic *E. coli* encoding Shiga toxin (stable toxin or heat-labile toxin).

SUPPLEMENTAL REFERENCES

1 Kotloff KL, Nataro JP, Blackwelder WC, *et al.* Burden and aetiology of diarrhoeal disease in infants and young children in developing countries (the Global Enteric Multicenter Study, GEMS): A prospective, case-control study. *Lancet* 2013; **382**: 209–22.

2 Kotloff KL, Nasrin D, Blackwelder WC, *et al.* The incidence, aetiology, and adverse clinical consequences of less severe diarrhoeal episodes among infants and children residing in low-income and middle-income countries: a 12-month case-control study as a follow-on to the Global Enteric Multicenter St. *Lancet Glob Heal* 2019; **7**: e568–84.

3 Blackwelder WC, Biswas K, Wu Y, *et al.* Statistical methods in the Global Enteric Multicenter Study (GEMS). *Clin Infect Dis* 2012. DOI:10.1093/cid/cis788.

4 Liu J, Platts-Mills JA, Juma J, *et al.* Use of quantitative molecular diagnostic methods to identify causes of diarrhoea in children: a reanalysis of the GEMS case-control study. *Lancet* 2016; **388**: 1291–301.

5 Platts-Mills JA, Liu J, Rogawski ET, *et al.* Use of quantitative molecular diagnostic methods to assess the aetiology, burden, and clinical characteristics of diarrhoea in children in low-resource settings: a reanalysis of the MAL-ED cohort study. *Lancet Glob Heal* 2018. DOI:10.1016/S2214-109X(18)30349-8.
